# Inhibition of H3K9 Methyltransferase G9a Repressed Cell Proliferation and Induced Autophagy in Neuroblastoma Cells

**DOI:** 10.1371/journal.pone.0106962

**Published:** 2014-09-08

**Authors:** Xiao-Xue Ke, Dunke Zhang, Shunqin Zhu, Qingyou Xia, Zhonghuai Xiang, Hongjuan Cui

**Affiliations:** State Key Laboratory of Silkworm Genome Biology, Southwest University, Chongqing, China; National University of Singapore, Singapore

## Abstract

Histone methylation plays an important role in gene transcription and chromatin organization and is linked to the silencing of a number of critical tumor suppressor genes in tumorigenesis. G9a is a histone methyltransferase (HMTase) for histone H3 lysine 9. In this study, we investigated the role of G9a in neuroblastoma tumor growth together with the G9a inhibitor BIX01294. The exposure of neuroblastoma cells to BIX01294 resulted in the inhibition of cell growth and proliferation, and BIX01294 treatment resulted in the inhibition of the tumorigenicity of neuroblastoma cells in NOD/SCID mice. Therefore, G9a may be a potential therapeutic target in neuroblastoma. Moreover, we found several specific characteristics of autophagy after BIX01294 treatment, including the appearance of membranous vacuoles and microtubule-associated protein light chain 3 (LC3B). Similar results were observed in G9a-knockdown cells. In conclusion, our results demonstrated that G9a is a prognostic marker in neuroblastoma, and revealed a potential role of G9a in regulating the autophagy signaling pathway in neuroblastoma.

## Introduction

Tumorigenesis is considered to be a multi-step process ranging from stages characterized by normal histological features to carcinoma features. Epigenetics has been recently defined as inheritable changes in gene expression not due to any alteration in the DNA sequence. Histone methylation is the fundamental epigenetic mechanism that regulates gene expression in cancers and is linked to the silencing of a number of critical tumor suppressor genes in tumorigenesis [1], [2]. Recently, G9a was reported to be a major H3K9me1 and H3K9me2 HMT in vivo [3]–[6], and several studies have identified the critical role that G9a plays in various biological processes, including embryo development, immune response, drug response and tumor cell growth [7]–[14]. Moreover, current evidence suggests that G9a promotes invasion and metastasis in lung cancer [13], and highly expressed G9a was observed in hepatocellular carcinomas [15]. Therefore, G9a may be a key regulator that serves as a potential therapeutic target during tumor formation.

In addition, autophagy is an evolutionarily conserved mechanism that involves the degradation of macromolecules, ribosomes, and organelles [16]. Autophagy is the primary intracellular catabolic process responsible for long-lived protein and organelle degradation and recycling, whereas the ubiquitin/proteasome system is the major cellular pathway responsible for short-lived protein degradation [17], [18]. The following four primary forms of autophagy have been described: macroautophagy (referred to here as autophagy), selective autophagy, microautophagy, and chaperone-mediated autophagy [19]–[21]. Autophagy serves as an adaptive response to cellular stress such as hypoxia and nutrient deprivation, which involves the synthesis of a double-membrane structure known as the phagophore. The phagophore ultimately elongates and closes to sequester cytoplasmic proteins and organelles, forming the autophagosome, and undergoes a stepwise maturation process [22]–[24]. Mammalian autophagy-related genes (ATG) participate in distinct steps of autophagy. For example, microtubule-associated protein light chain 3 (LC3B) undergoes lipidation and is recruited to the phagophore where it is essential for membrane elongation and closure [20].

Neuroblastoma is a common childhood malignant tumor of neural crest origin, arising in the sympathetic nervous system, and this condition accounts for approximately 10% of pediatric cancers and 15% of cancer-related deaths in children [25]–[31]. In this study, we investigated the role of G9a in neuroblastoma tumor growth together with BIX01294, which is a specific G9a inhibitor [32]–[34]. We provide experimental evidence supporting the role of G9a in the transcriptional regulation of autophagy in neuroblastoma cells. Pharmacological inhibition or RNA interference (RNAi) of G9a led to increased LC3B expression and autophagosome formation. Collectively, we identified G9a as a prognostic marker for survival in patients with neuroblastoma and a regulator of neuroblastoma cell growth, proliferation and autophagy. Our results suggest a novel potential role of G9a in the regulation of the autophagy signaling pathway in neuroblastoma.

## Materials and Methods

### Cell culture

The neuroblastoma cell line BE(2)-C was grown in a 1∶1 mixture of Dulbecco's modified Eagle's medium and Ham's nutrient mixture F12 (DMEM/F-12) (Life Technologies, Grand Island, NY, United States) supplemented with 10% fetal bovine serum (FBS) (Life Technologies). Other neuroblastoma cells (SK-N-AS, SK-N-DZ, SK-N-F1, and SHEP1) were cultured in Dulbecco's modified Eagle's medium (DMEM) (Life Technologies) plus 10% FBS. All of the cells were obtained from the American Type Culture Collection (ATCC, Manassas, VA, United States) and cultured at 37°C in a 5% CO_2_ humidified incubator.

### Cell proliferation and cell cycle assays

The G9a inhibitor BIX01294 (B9311, Sigma-Aldrich, St. Louis, MO, United States) was dissolved in water. The cells were grown to 60–70% confluence and treated with the indicated concentrations of BIX01294 [35]; cells treated with water were used as a control. After treatment, the adherent and floating cells were pooled at different time points, collected by centrifugation, and then washed once with ice-cold PBS. The sample obtained was analyzed with the TC10 Automated Cell Counter (Bio-Rad, Hercules, CA, United States), and cell counting was determined using trypan blue dye (#145-0021, Bio-Rad). The samples for the cell cycle assay were fixed with 70% ethanol, stained with PI, and analyzed by flow cytometry (BD FACSVerse, BD BioSciences, San Jose, CA, United States). The data were analyzed with CellQuest Pro software (BD BioSciences). The cell growth curve was detected by CCK-8 (CK04-05, DOJINDO, Kamimashiki gun, Kumamoto, Japan).

### Immunofluorescence analysis

For immunofluorescent staining, the cells were grown on coverslips. After treatment with BIX01294, the cells were washed with PBS and fixed in 4% paraformaldehyde in PBS for 20 min at room temperature. Then, the cells were permeabilized with 0.3% Triton X-100 for 5 min. The cells were blocked with 5% milk, incubated with a primary antibody, and then incubated with the appropriate secondary antibody. The primary antibodies were used at a dilution of 1∶500 for the rabbit monoclonal antibody against LC3B (Cell Signaling Technologies, Danvers, MA, United States) and 1∶2000 for the mouse monoclonal antibody against α-tubulin (clone B-5-1-2, Sigma-Aldrich). Alexa Fluor 488 Goat Anti-Rabbit IgG (H+L) and Alexa Fluor 594 Goat Anti-Mouse IgG (H+L) (Life Technologies, Carlsbad, CA, United States) were used as secondary antibodies. In total, 300 nM DAPI in PBS was used for nuclear staining.

### Lentiviral infection

The lentiviral human G9a shRNA constructions were obtained from Open Biosystems (Thermo Fisher Scientific, Pittsburgh, PA, United States). The lentiviruses were produced by co-transfection with an shRNA-expressing vector or GFP shRNA. The lentiviral constructs were transfected into 293FT packaging cells using Lipofectamine 2000 (Life Technologies). Virus-containing supernatants were harvested and titered and then were used to infect target cells with 4 µg/ml polybrene (sc-134220, Santa Cruz, Dallas, Texas, United States). One day after the final round of infection, the cells were cultured in the presence of 2 µg/ml puromycin (A1113803, Life Technologies) for 3 days, and the drug-resistant cells were pooled.

### Western blot analysis

Western blot analysis was performed with the primary antibodies including anti-G9a (1∶500, Santa Cruz), anti-α-tubulin (1∶2000, Sigma-Aldrich), anti-H3K9me2 (1∶500, Abcam, Cambridge, MA, United States), anti-CDK2 (1∶500, Santa Cruz), anti-CDK4 (1∶500, Santa Cruz), anti-CDK6 (1∶500, Santa Cruz), anti-CyclinD1 (1∶500, Santa Cruz), anti-CyclinE (1∶500, Abcam), anti-LC3B (1∶500, Cell Signaling Technologies), and the autophagy antibody sampler kit (1∶500, Cell Signaling Technologies). Horseradish peroxidase-conjugated goat anti-mouse (1∶20000), goat anti-rabbit (1∶20000), and rabbit anti-goat (1∶10000) immunoglobulin G (IgG; KPL, Gaithersburg, Maryland, United States) were used as secondary antibodies.

### Soft agar clonogenic assay

In total, 1×10^3^–2.5×10^3^ cells were mixed with 0.3% Noble agar in growth medium and plated into six-well plates containing a solidified bottom layer (0.6% Noble agar in growth medium). The colonies were photographed after 14 to 21 days and recorded.

### *In vivo* tumorigenic assay

Six age-matched non-obese diabetic severe combined immunodeficient (NOD/SCID) female mice (4 weeks old) were used in this study. The mice were housed with 2–3 animals per cage and maintained under Specific Pathogen-Free (SPF) conditions. For the experimental tumorigenesis assays, 1 × 10^6^–3×10^6^ cells were resuspended in 100 µl DMEM and injected subcutaneously into both flanks of each mouse. After 1 week of tumor growth, the mice were randomly divided into two groups. One group was injected intraperitoneally with BIX01294 at 4 mg/kg (mice body weight), and the other group was injected with water as a control. Tumor growth was measured by caliper measurement, and tumor volume was calculated with the formula 4/3пr^3^, where r is the radius of the tumor. Four weeks after injection, the tumors were removed and weighed.

### Ethics statement

This study was conducted in accordance with the approved guidelines. The protocol was pre-approved by the Institutional Animal Care and Use Committee of Southwest University. All efforts were made to minimize the suffering of the animals.

### Patient data analysis

Patient data and gene expression datasets were obtained from the Oncogenomics Section Data Center (http://pob.abcc.ncifcrf.gov/cgi-bin/JK) and R2: microarray analysis and visualization platform (http://hgserver1.amc.nl/cgi-bin/r2/main.cgi). All prognosis analyses were conducted online, and all data and P values (log-rank test) were downloaded. Kaplan–Meier analysis and the resulting survival curves were performed using GraphPad Prism (version 6.0). All cutoff values for separating high and low expression groups were determined by the online R2 or Oncogenomics database algorithm [36], [37].

### Statistical analysis

All observations were confirmed by at least three independent experiments. Quantitative data are expressed as the mean ±SD. Two-tailed Student's t-test was performed for paired samples. P<0.05 was considered statistically significant.

## Results

### G9a expression in neuroblastoma is associated with poor prognosis

**Figure 1:**
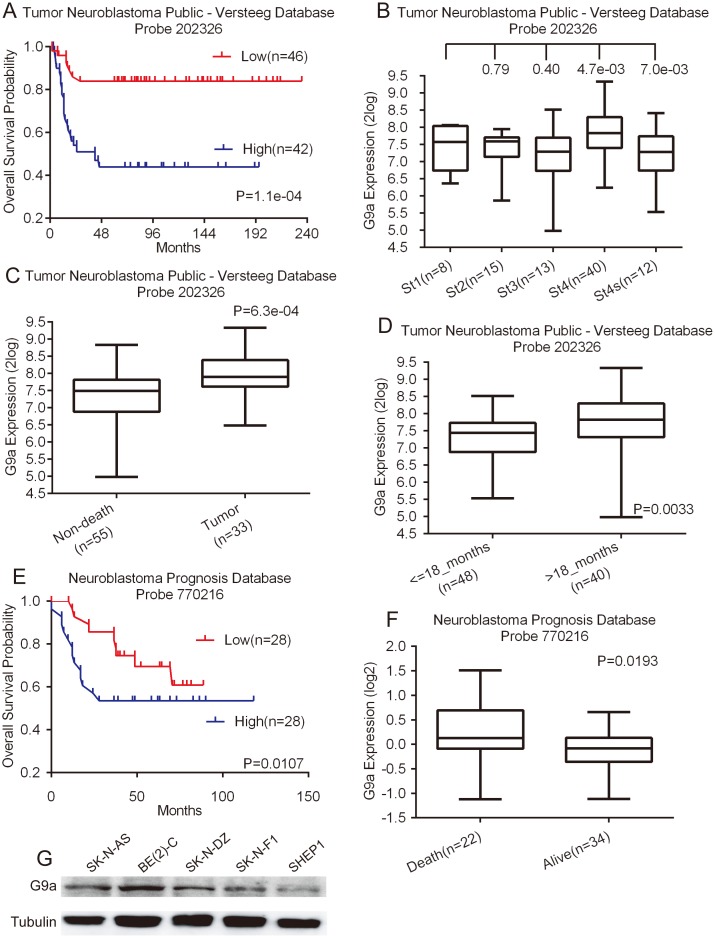
High G9a expression is a prognostic indicator of poor survival in neuroblastoma patients. A, Kaplan–Meier analysis of progression-free survival for the Versteeg database with the log rank test P value indicated. B, box plot of G9a expression levels in stage (ST) 1–4S tumors. C, box plot of G9a expression levels in tumors from patients in the non-death and tumor-caused-death groups. D, box plot of G9a expression levels in tumors from patients in the <18-month and >18-month groups. E, Kaplan–Meier analysis of progression-free survival for the Neuroblastoma Prognosis Database with the log rank test P value indicated. F, box plot of G9a expression levels in tumors from dead and alive groups. G, western blot analysis of G9a expression in five neuroblastoma cell lines. For the data in A, the G9a cutoff value of 7.62 was used to separate the patients into high and low G9a expression groups. The data in B (ST1 vs. ST2, ST2 vs. ST3, ST3 vs. ST4, and ST4 vs. ST4S), C, D, and F were analyzed using two-tailed student's t-test with the P values indicated. For the data in E, the G9a cutoff value of 0 was used to separate the patients into high and low G9a expression groups.

To investigate the possibility of G9a as a prognostic marker in neuroblastoma, we conducted a microarray-based search using the Tumor Neuroblastoma public - Versteeg database [36], which is available from the online R2: microarray analysis and visualization platform. The Versteeg database contains a cohort of 88 patients with neuroblastoma representative of various tumor stages and genetic alterations. Kaplan–Meier analysis of progression-free survival for the Versteeg database showed that high G9a expression was strongly associated with a poor outcome, whereas low G9a expression was correlated with good overall survival (Fig. 1A). Moreover, G9a expression was different in various tumor stages (Fig. 1B), and G9a expression increased significantly in stage 4 tumors compared to stages 3 and 4S (Fig. 1B). Then, we examined the correlation of G9a expression levels with patient cause of death using data available from the Versteeg database. The result revealed that the G9a expression level was significantly higher in tumor-caused death than non-death group (Fig. 1C).

A younger patient at diagnosis typically has a good prognosis [38]; therefore, we examined the correlation of G9a expression levels with patient age at diagnosis using information available from the Versteeg database. We chose the cutoff at 18 months of age because it provided the most significant P value for prognostic evaluation [39]. The younger age group expressed significantly lower levels of G9a than the older age group (Fig. 1D), showing a correlation of low G9a expression with younger age at diagnosis. Overall, our data suggest that higher levels of G9a predict poor prognosis in neuroblastoma. We confirmed that the high expression of G9a is prognostic of unfavorable outcomes with the Neuroblastoma Prognosis Database available from the online Oncogenomics database [37], which includes a cohort of 56 neuroblastoma patients (Fig. 1E and F). Together, our analyses of two independent microarray databases indicate that G9a is a prognostic marker in neuroblastoma.

Then, we confirmed the G9a expression levels in five neuroblastoma cell lines, SK-N-AS, BE(2)-C, SK-N-DZ, SK-N-F1, and SHEP1 (Fig. 1G). We found that G9a is highly expressed in all five cell lines, indicating that G9a is commonly expressed in neuroblastoma.

### G9a inhibition represses neuroblastoma cell growth and proliferation

**Figure 2:**
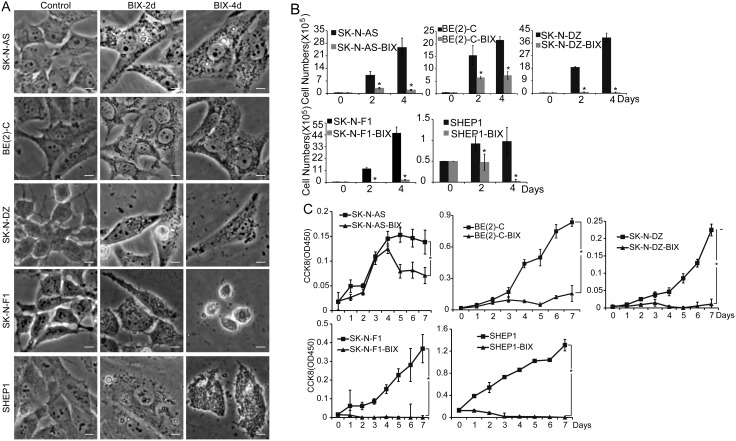
Inhibition of G9a represses neuroblastoma cell growth and proliferation. A, morphologic examination of five neuroblastoma cells treated with BIX01294 or water for 2 and 4 days. Scale bars, 5 µm. B, neuroblastoma cells were either treated with 5 µM BIX01294 or water for 2 days and 4 days, respectively, then analyzed for cell counting with the TC10 Automated Cell Counter, error bars, SD, n = 5. Statistical analysis was performed using two-tailed student's t-test, *p≤0.01. C, neuroblastoma cells were either treated with 5 µM BIX01294 or water and then analyzed for cell growth by the CCK8 assay. Each value represents the average obtained from five independent experiments; error bars, SD. Statistical analysis was performed using two-tailed student's t-test, *p≤0.01.

**Figure 3:**
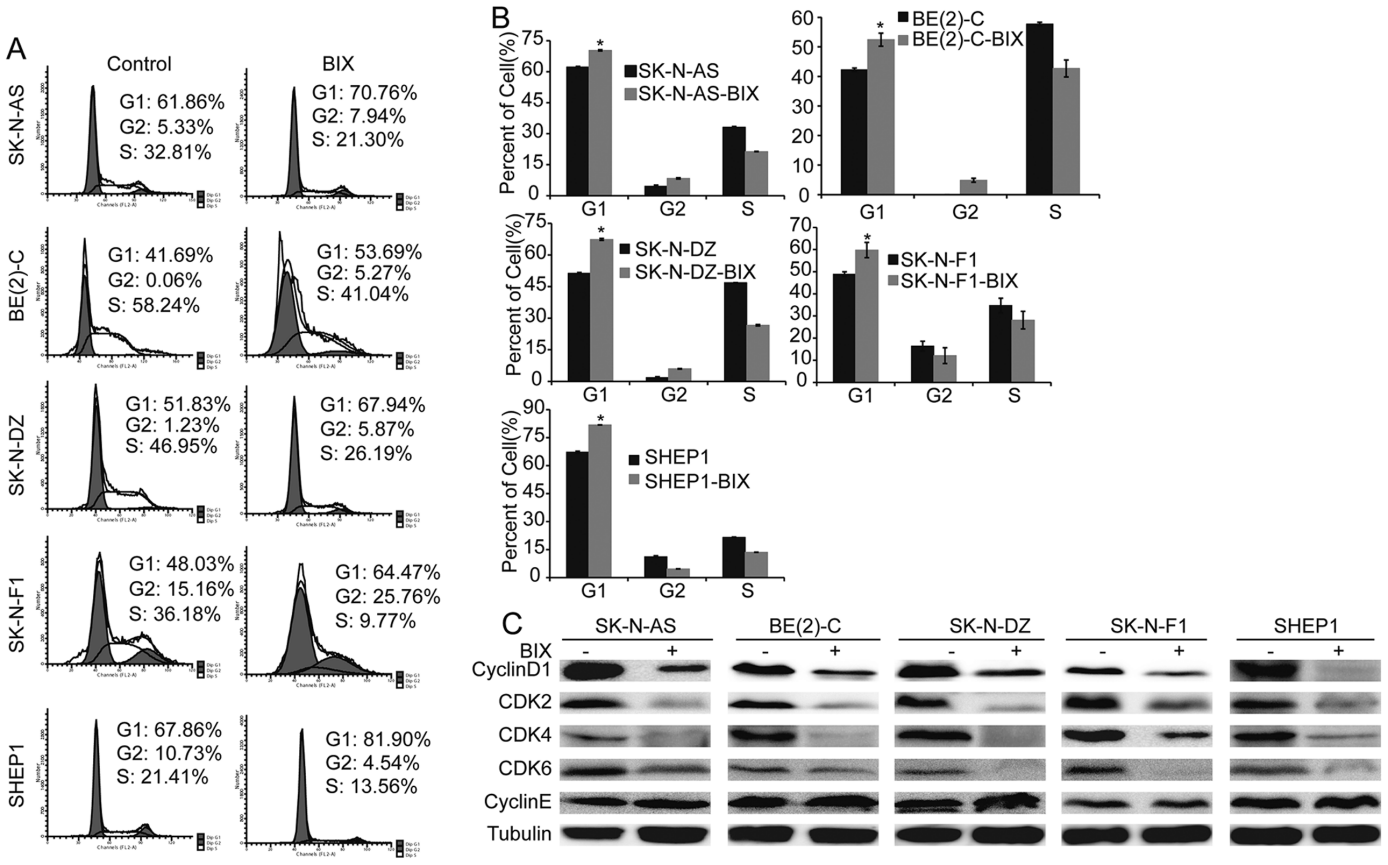
Inhibition of G9a induces cell cycle arrest in G1 phase. A and B, neuroblastoma cells were either treated with 5 µM BIX01294 or water for 2 days and analyzed for the cell cycle by flow cytometry. Each column represents the average obtained from three independent experiments; error bars, SD. Statistical analysis was performed using two-tailed student's t-test, *p≤0.01. C, western blot analysis of cyclins and CDKs associated with G1 phase in neuroblastoma cells treated with BIX01294 or water for 2 days. α-Tubulin levels are shown as the loading control.

We next examined the functional consequence of high expression of G9a in neuroblastoma cells. The SK-N-AS, BE(2)-C, SK-N-DZ, SK-N-F1, and SHEP1 neuroblastoma cell lines were treated with BIX01294, a specific inhibitor of G9a. As shown in Figure S1A, G9a inhibition suppressed BE(2)-C cell proliferation in a dose-dependent manner. All five cell lines were very sensitive to BIX01294 treatment, which dramatically repressed cell proliferation after 4 days of treatment (Fig. S1B). This result was confirmed by the cell counting kit-8 (CCK8) assay (Fig. 2C), and the cell number was highly decreased after treatment with 5 µM BIX01294 (Fig. 2B). We further examined the cell cycle in neuroblastoma cells. After drug treatment, the number of cells in the G1 phase increased and the number of cells in the S phase decreased in SK-N-AS, BE(2)-C, SK-N-DZ, SK-N-F1, and SHEP1 cultures (Fig. 3A and B), suggesting that G9a inhibition caused by BIX01294 treatment induced cell cycle arrest in G1 phase. Western blot analysis showed that G9a inhibition led to a marked down-regulation of CyclinD1, CDK4 and CDK6, which are collectively required for the cell cycle progression from G1 to the S phase [40], [41]. Moreover, G9a inhibition led to an obvious down-regulation of CDK2 and no significant change in CyclinE, which are required for the cell cycle progressions from S to G2 [40], [41] (Fig. 3C). These results showed that G9a inhibition in neuroblastoma cells completely blocked cell proliferation, arresting the cells in the G1 phase. Together, these results suggest that G9a plays an important role in neuroblastoma cell growth and proliferation.

### Inhibition of G9a induces neuroblastoma cell autophagy

**Figure 4:**
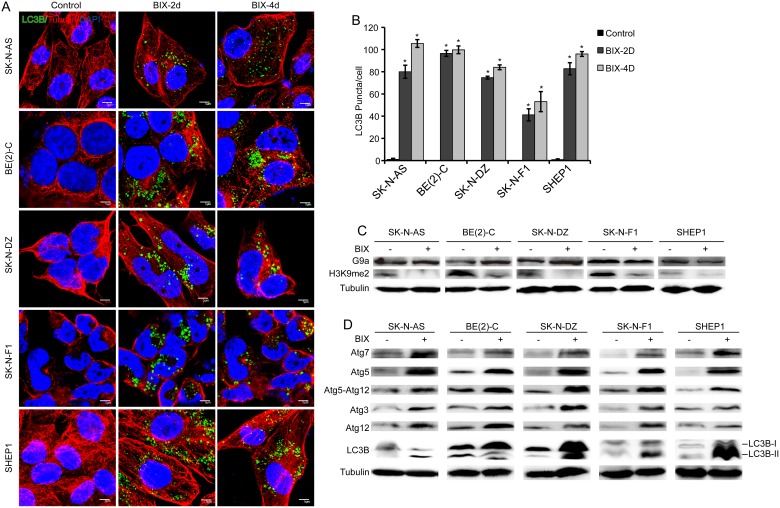
Inhibition of G9a induces autophagy in neuroblastoma cells. A, immunofluorescence analysis of five neuroblastoma cells treated with BIX01294 or water for 2 and 4 days. Scale bars, 5 µm. B, Statistical analysis of LC3B puncta in neuroblastoma cells treated with BIX01294 or water for 2 and 4 days. Each column represents the average obtained from three independent experiments; error bars, SD. Statistical analysis was performed using two-tailed student's t-test, *p≤0.01. C, western blot analysis of G9a function in neuroblastoma cells treated with BIX01294 or water for 2 days. D, western blot analysis of autophagy-related genes in neuroblastoma cells treated with BIX01294 or water for 2 days. α-Tubulin levels are shown as the loading control.

To elucidate the contribution of G9a to the inhibition of neuroblastoma cell proliferation, we conducted immunofluorescence staining. After 2 days of treatment, an autophagosome-like structure was observed in neuroblastoma cells, and this structure developed in a time-dependent manner after 4 days of treatment (Fig. 2A). Next, we investigated LC3B expression, which is a marker of autophagy [42]–[44]. As shown in Fig. 4A and B, we found abundant LC3B expression in cells with G9a inhibition induced by BIX01294 treatment. To gain insight into the molecular mechanism underlying G9a inhibition-induced LC3B expression, we analyzed the protein expression of all five neuroblastoma cells. BIX01294 treatment had no significant effect on G9a expression in neuroblastoma cells, but it led to a marked down-regulation of H3K9me2, which was reported to be a major product in G9a-mediated H3K9 methylation [6] (Fig. 4C). These findings indicate that the inhibitor BIX01294 had no effect on G9a protein expression, but it suppressed the methyltransferase function of G9a. Moreover, we investigated whether BIX01294 treatment induces the expression of genes known to participate in autophagosome formation. The Western blot assay revealed that the expression of ATGs and LC3B was markedly up-regulated after treatment (Fig. 4D). These results indicate that the loss of G9a function induces autophagy and autophagosome formation in neuroblastoma cells.

### Inhibition of G9a decreases tumorigenicity of neuroblastoma cells

**Figure 5:**
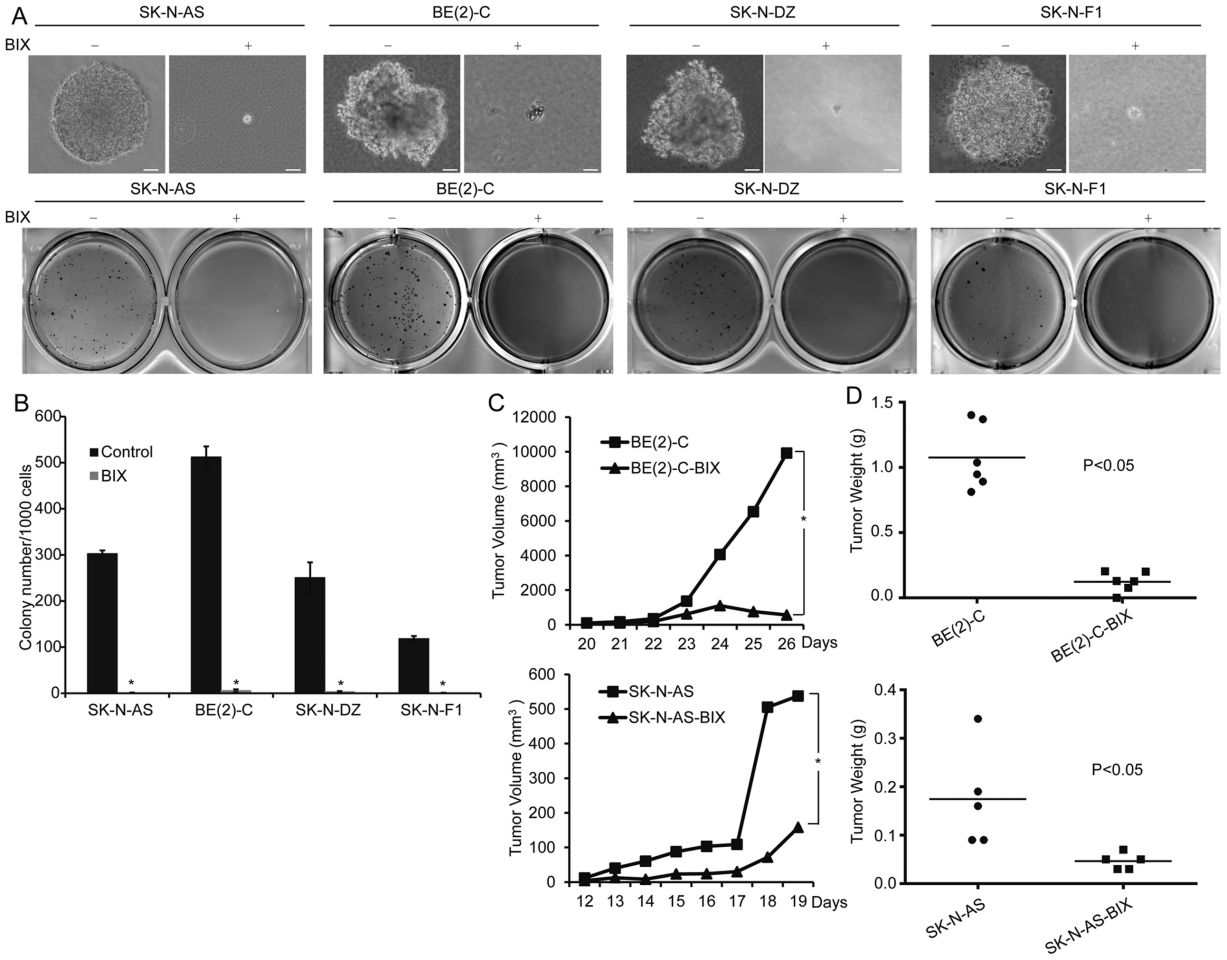
Inhibition of G9a decreases the tumorigenicity of neuroblastoma cells. A, neuroblastoma cells were plated at 1×10^3^ to 2.5×10^3^ cells per well in six-well culture plates. After 14 to 21 days of culture, soft agar colonies developed with cells treated with water. As shown, the cells treated with 5 µM BIX01294 were observed to give rise to small and scanty colonies in soft agar. Scale bars, 50 µm. B, colonies that were larger than 0.5 mm or that contained more than 50 cells were recorded. Each column represents the average obtained from three independent experiments; error bars, SD. Statistical analysis was performed using two-tailed student's t-test, *p≤0.01. C, tumor growth in NOD/SCID mice injected with the indicated neuroblastoma cells as measured by caliper measurements. D, scatter plot of xenograft tumor weight with horizontal lines indicating the mean per group. The data were analyzed with 2-tailed Student t test, and the P value is indicated.

To evaluate the role of G9a for the tumorigenicity of neuroblastoma cells, we first examined this phenomenon using soft agar clonogenic assays. The BE(2)-C cells were plated at 1×10^3^ cells per well (six-well culture plates). After plating, immediate examination under a microscope revealed mostly individual cells and colonies, defined as a collection of more than 50 cells appearing after 14 to 21 days. The BE(2)-C cells treated with 5 µM BIX01294 were observed to give rise to small and scanty colonies in soft agar compared to the cells treated with water. Similar results were also obtained with several other neuroblastoma cell lines, including SK-N-AS, SK-N-DZ, and SK-N-F1 cells (Fig. 5A and B). Together, these results indicate that G9a inhibition prevents the clonogenic activity of neuroblastoma cells. Next, we examined the effect of G9a on the ability of neuroblastoma cells to induce tumors in immunodeficient mice. The neuroblastoma cell lines BE(2)-C and SK-N-AS were injected subcutaneously into the flanks of NOD/SCID mice. After 1 week of tumor growth, BIX01294 was injected intraperitoneally at 4 mg/kg. NOD/SCID mice were injected with water as a control. The mice without BIX01294 treatment developed large tumor masses after three weeks, whereas BIX01294 injection significantly diminished the tumorigenic activity of neuroblastoma cells during the same time period (Fig. 5C and D). All of the above results demonstrated that G9a inhibition decreases the tumorigenicity of neuroblastoma cells.

### Downregulation of G9a represses cell proliferation and induces autophagy in neuroblastoma

**Figure 6:**
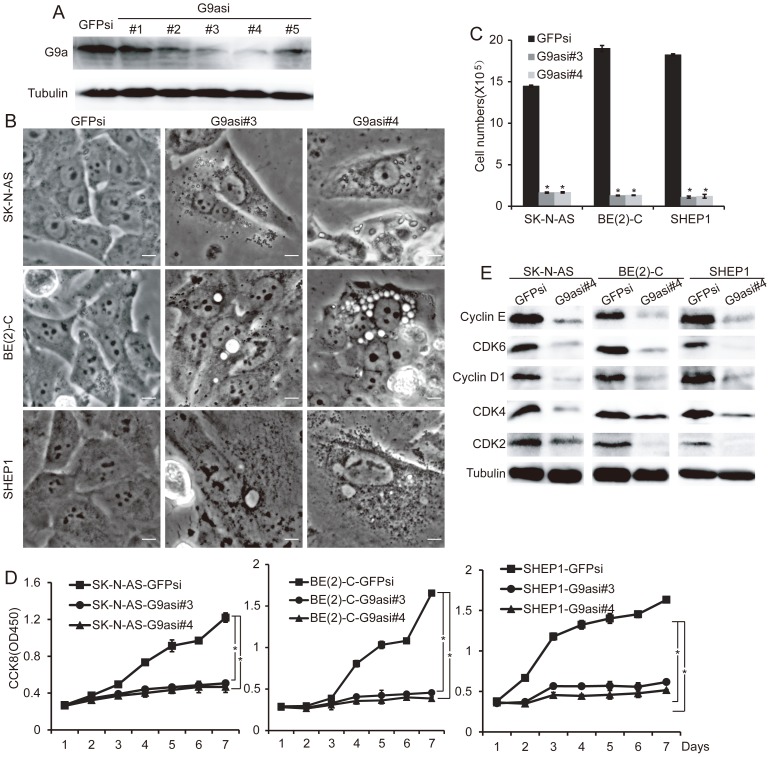
Downregulation of G9a represses neuroblastoma cell growth and proliferation. A, western blot analysis of G9a in neuroblastoma cells BE(2)-C expressing GFPsi or individual G9asi sequences. α-Tubulin levels are shown as the loading control. B, morphological examination of three neuroblastoma cells expressing GFPsi, G9asi#3 or G9asi#4, respectively. The cells expressing GFPsi are shown as the biological controls. Scale bar, 5 µm. C, neuroblastoma cells expressing GFPsi, G9asi#3 or G9asi#4 were analyzed for cell counting using the TC10 Automated Cell Counter, error bars, SD, n = 5. Statistical analysis was performed using two-tailed student's t-test, *p≤0.01. D, proliferation assays of three neuroblastoma cells expressing GFPsi, G9asi#3 or G9asi#4, error bars, SD, n = 5. E, western blot analysis of cyclins and CDKs related to the G1 phase in neuroblastoma cells with G9a knockdown. α-Tubulin levels are shown as the loading control.

**Figure 7:**
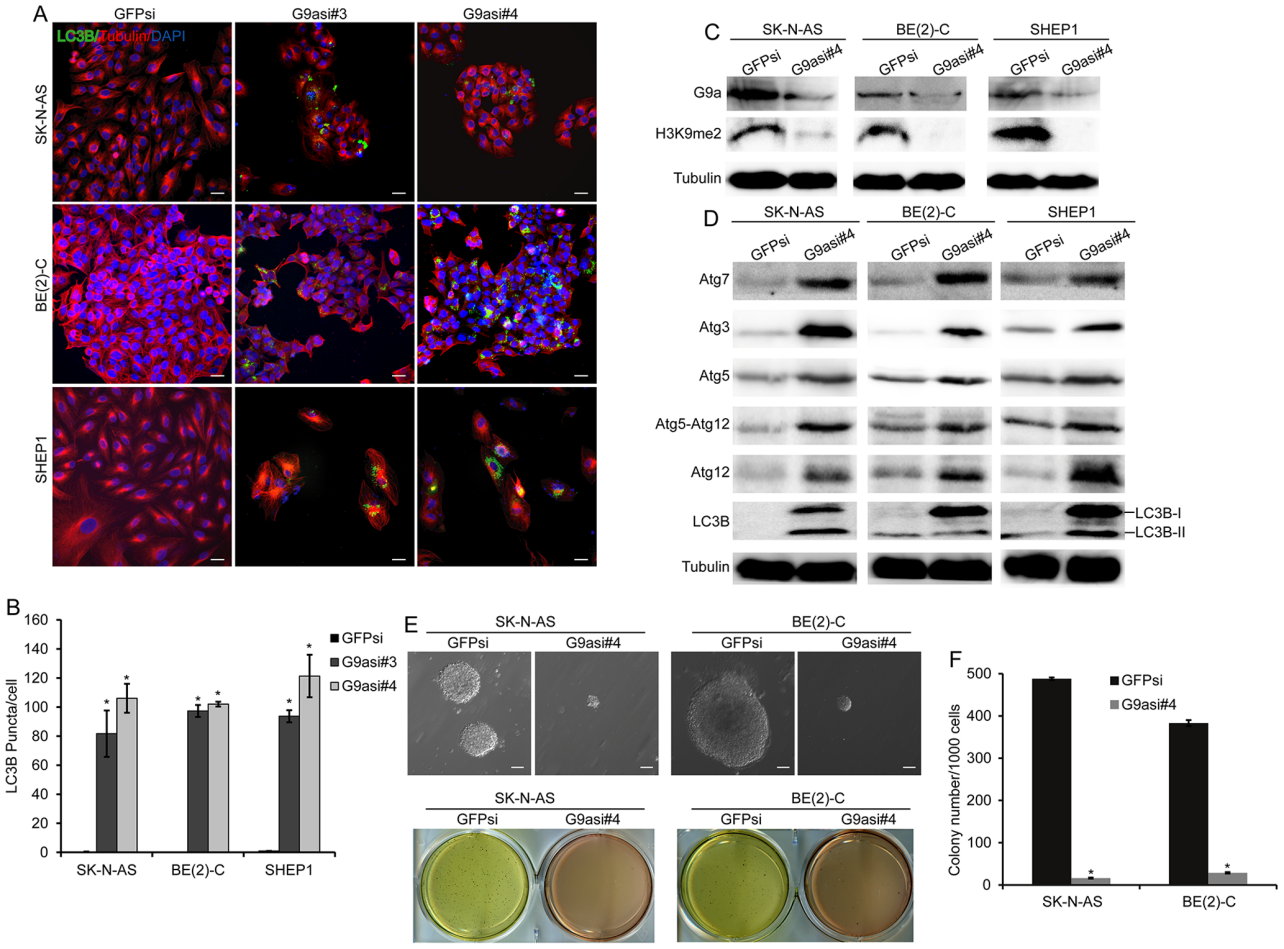
Downregulation of G9a induces autophagy and decreases tumorigenicity in neuroblastoma cells. A, immunofluorescence analysis of three neuroblastoma cells expressing GFPsi, G9asi#3 or G9asi#4. Scale bar, 5 µm. B, statistical analysis of LC3B puncta in neuroblastoma cells expressing GFPsi, G9asi#3 or G9asi#4. Each column represents the average obtained from three independent experiments; error bars, SD. Statistical analysis was performed using two-tailed student's t-test, *p≤0.01. C and D, western blot analysis of G9a function (C) and autophagy-related genes (D) in neuroblastoma cells expressing GFPsi or G9asi#4. α-Tubulin levels are shown as the loading control. Cells expressing GFPsi are shown as the biological control. E, neuroblastoma cells were plated at 1×10^3^ cells per well in six-well culture plates. After 14 to 21 days of culture, soft agar colonies developed with cells expressing GFPsi. As shown, the cells with G9a knockdown were observed to give rise to small and scanty colonies in soft agar, Scale bars, 50 µm. F, Colonies that were larger than 0.5 mm or that contained more than 50 cells were recorded. Each column represents the average obtained from three independent experiments; error bars, SD. Statistical analysis was performed using two-tailed student's t-test, *p≤0.01.

In addition to the inhibition of G9a by BIX01294, we knocked down G9a in the three neuroblastoma cell lines SK-N-AS, BE(2)-C and SHEP1. We employed five lentiviral constructs expressing short hairpin RNA sequences against human G9a (G9asi), and three of them significantly reduced G9a expression in the neuroblastoma cells (Figure 6A). We performed the subsequent experiments with neuroblastoma cells expressing either G9asi#3 or G9asi#4. Biochemically, we noted that G9a knockdown in the three neuroblastoma cell lines induced cell growth arrest, which was determined by morphology and proliferation assays (Fig. S1C, Fig. 6C and D). Western blot analysis showed that G9a knockdown also led to a marked down-regulation of CyclinD1, CDK4 and CDK6 in the three neuroblastoma cell lines (Fig. 6E, Fig. S2A). These results showed that G9a down-regulation represses neuroblastoma cell growth and proliferation. Furthermore, we noted that G9a knockdown in three neuroblastoma cell lines also induced autophagosome formation and LC3B expression as determined by morphology and immunofluorescence assays (Fig. 6B, Fig. 7A and B). In addition, G9a knockdown in the neuroblastoma cells markedly inhibited the H3K9me2 level and up-regulated the ATGs and LC3B expression levels (Fig. 7C and D, Fig. S2B and C). These findings demonstrate that the down-regulation of G9a-induced autophagy in neuroblastoma cells. Moreover, we examined the tumorigenicity of the G9a-knockdown cells. The result showed that the neuroblastoma cells that underwent G9a knockdown failed to form large colonies in soft agar (Fig. 7E and F, Fig. S2D and E). Collectively, these results indicate that the knockdown of G9a in neuroblastoma cells represses cell proliferation, decreases tumorigenicity, and induces autophagy.

## Discussion

The intent of this paper was to characterize the role of the H3K9 methyltransferase G9a in neuroblastoma cell growth and tumor development. In this study, we investigated the effect of G9a together with its novel small molecular inhibitor BIX01294 in neuroblastoma cells. Specifically, we sought to determine the function of G9a in cell growth, tumorigenicity and histone methylation in neuroblastoma cells. Our results demonstrated G9a expression in all five neuroblastoma cell lines, SK-N-AS, BE(2)-C, SK-N-DZ, SK-N-F1, and SHEP1, suggesting a common expression of G9a in neuroblastoma. Furthermore, our results, based on two independent microarray databases, indicated that higher levels of G9a predict poor neuroblastoma prognosis. In addition, we found that tumors from older patients (>18 months) expressed G9a mRNA at significantly higher levels than tumors from younger patients (<18 months), suggesting that neuroblastomas initiated during early embryonic development are more likely to express lower levels of G9a.

In addition, our data indicate that the treatment of neuroblastoma cells with BIX01294 significantly inhibited cell growth and proliferation. The results from this study further demonstrated that BIX01294 treatment suppressed the tumorigenicity of neuroblastoma cells both in vitro and in vivo. It was unclear how G9a inhibition caused by this small molecular agent promotes growth suppression through cell death or the inhibition of cell division [45], [46]. To determine the mechanism by which the growth-inhibitory effects of G9a are overcome, flow cytometry experiments were performed. The results demonstrated that the cell cycle was altered after drug treatment. For example, the number of cells in G1 phase was increased and the number of cells in S phase was decreased. Our findings based on BIX01294-induced inhibition of G9a suggest that G9a may have an oncogenic function in the pathogenesis of neuroblastoma.

Interestingly, we found several specific characteristics of autophagy after BIX01294 treatment, including the appearance of membranous vacuoles and the accumulation of LC3B in autophagosome [47]–[49]. Similar results were observed in G9a-knockdown cells. Recently, it was reported that BIX01294 induces apoptosis in three neuroblastoma cell lines (LA1-55n, IMR-5, and NMB) [50], which was different from our findings. Thus, it was particularly interesting to identify the molecular mechanism of the regulation mediated by G9a in neuroblastoma growth. BIX01294 may induce autophagy-associated cell death in MCF-7 cells [51]; therefore, we speculate that G9a is involved in a novel regulatory pathway of autophagy in neuroblastoma. Herein, we provide evidence that the pharmacologic inhibition or genetic depletion of the histone methyltransferase G9a leads to the suppression of cell proliferation, the formation of autophagosome-like structures, the aggregation of LC3B and the expression of ATGs. Moreover, our data suggest that G9a represses the level of histone H3K9me2, leading to increased expression of LC3B and ATGs to support the process of autophagy. Taken together, these data indicate a role for G9a in the epigenetic regulation of autophagy and suggest that either pharmacologic manipulation of G9a or the genetic regulation of G9a may impact the autophagy process.

In conclusion, our results define the critical function of G9a in cell growth and tumorigenicity and suggest that G9a is involved in a novel regulatory pathway of autophagy in neuroblastoma.

## Supporting Information

Figure S1 **Inhibition of G9a suppresses neuroblastoma cell proliferation.** A, neuroblastoma BE(2)-C cells were treated with BIX01294 for 48 h at concentrations of 1, 2.5, 5, 7.5, and 10 µM. Proliferation was determined by trypan blue exclusion to differentiate between dead and live cells. Error bars, SD, n = 5. Statistical analysis was performed using two-tailed student's t-test, *p≤0.01. B, morphologic examination of five neuroblastoma cells treated with 5 µM BIX01294 or water for 2 and 4 days. Scale bars, 20 µm. C, morphological examination of three neuroblastoma cell lines express GFPsi, G9asi#3 or G9asi#4. Cells expressing GFPsi are shown as the biological control. Scale bar, 20 µm.
(TIF)

Figure S2 **Downregulation of G9a represses neuroblastoma cell proliferation and tumorigenicity and induces autophagy.** A, B and C, western blot analysis of cyclins and CDKs associated with the G1 phase (A), G9a function (B) and autophagy-related genes (C) in neuroblastoma cells expressing GFPsi or G9asi#3. α-Tubulin levels are shown as the loading control. Cells expressing GFPsi are shown as the biological control. D, neuroblastoma cells were plated at 1×10^3^ cells per well in six-well culture plates. After 14 to 21 days of culture, soft agar colonies grown with cells expressing GFPsi. As shown, the cells with G9a knockdown were observed to give rise to small and scanty colonies in soft agar, Scale bars, 50 µm. E, colonies that were larger than 0.5 mm or that contained more than 50 cells were recorded. Each column represents the average obtained from three independent experiments; error bars, SD. Statistical analysis was performed using two-tailed student's t-test, *p≤0.01.
(TIF)

## References

[1] Bachman KE, Park BH, Rhee I, Rajagopalan H, Herman JG, et al (2003) Histone modifications and silencing prior to DNA methylation of a tumor suppressor gene. Cancer cell 3: 89–95. 12559178 10.1016/s1535-6108(02)00234-9

[2] Calcagno DQ, Gigek CO, Chen ES, Burbano RR, Smith Mde A (2013) DNA and histone methylation in gastric carcinogenesis. World journal of gastroenterology: WJG 19: 1182–1192. 23482412 10.3748/wjg.v19.i8.1182 PMC3587474

[3] Tachibana M, Sugimoto K, Nozaki M, Ueda J, Ohta T, et al (2002) G9a histone methyltransferase plays a dominant role in euchromatic histone H3 lysine 9 methylation and is essential for early embryogenesis. Genes & development 16: 1779–1791. 12130538 10.1101/gad.989402 PMC186403

[4] Peters AH, Kubicek S, Mechtler K, O'Sullivan RJ, Derijck AA, et al (2003) Partitioning and plasticity of repressive histone methylation states in mammalian chromatin. Molecular cell 12: 1577–1589. 14690609 10.1016/s1097-2765(03)00477-5

[5] Rice JC, Briggs SD, Ueberheide B, Barber CM, Shabanowitz J, et al (2003) Histone methyltransferases direct different degrees of methylation to define distinct chromatin domains. Molecular cell 12: 1591–1598. 14690610 10.1016/s1097-2765(03)00479-9

[6] Shinkai Y, Tachibana M (2011) H3K9 methyltransferase G9a and the related molecule GLP. Genes & development 25: 781–788. 21498567 10.1101/gad.2027411 PMC3078703

[7] Feldman N, Gerson A, Fang J, Li E, Zhang Y, et al (2006) G9a-mediated irreversible epigenetic inactivation of Oct-3/4 during early embryogenesis. Nature cell biology 8: 188–194. 16415856 10.1038/ncb1353

[8] Ohhata T, Tachibana M, Tada M, Tada T, Sasaki H, et al (2004) X-inactivation is stably maintained in mouse embryos deficient for histone methyl transferase G9a. Genesis 40: 151–156. 15493016 10.1002/gene.20077

[9] El Gazzar M, Yoza BK, Chen X, Hu J, Hawkins GA, et al (2008) G9a and HP1 couple histone and DNA methylation to TNFalpha transcription silencing during endotoxin tolerance. The Journal of biological chemistry 283: 32198–32208. 18809684 10.1074/jbc.M803446200 PMC2583293

[10] Ma DK, Chiang CH, Ponnusamy K, Ming GL, Song H (2008) G9a and Jhdm2a regulate embryonic stem cell fusion-induced reprogramming of adult neural stem cells. Stem cells 26: 2131–2141. 18535151 10.1634/stemcells.2008-0388 PMC4059405

[11] Thomas LR, Miyashita H, Cobb RM, Pierce S, Tachibana M, et al (2008) Functional analysis of histone methyltransferase g9a in B and T lymphocytes. Journal of immunology 181: 485–493. 10.4049/jimmunol.181.1.485 PMC2497432 18566414

[12] Goyama S, Nitta E, Yoshino T, Kako S, Watanabe-Okochi N, et al (2010) EVI-1 interacts with histone methyltransferases SUV39H1 and G9a for transcriptional repression and bone marrow immortalization. Leukemia 24: 81–88. 19776757 10.1038/leu.2009.202

[13] Chen MW, Hua KT, Kao HJ, Chi CC, Wei LH, et al (2010) H3K9 histone methyltransferase G9a promotes lung cancer invasion and metastasis by silencing the cell adhesion molecule Ep-CAM. Cancer research 70: 7830–7840. 20940408 10.1158/0008-5472.CAN-10-0833

[14] Dong C, Wu Y, Yao J, Wang Y, Yu Y, et al (2012) G9a interacts with Snail and is critical for Snail-mediated E-cadherin repression in human breast cancer. The Journal of clinical investigation 122: 1469–1486. 22406531 10.1172/JCI57349 PMC3314447

[15] Wu H, Zhang H, Wang P, Mao Z, Feng L, et al (2013) Short-Form CDYLb but not long-form CDYLa functions cooperatively with histone methyltransferase G9a in hepatocellular carcinomas. Genes, chromosomes & cancer 52: 644–655. 23629948 10.1002/gcc.22060

[16] Berardi DE, Campodonico PB, Diaz Bessone MI, Urtreger AJ, Todaro LB (2011) Autophagy: friend or foe in breast cancer development, progression, and treatment. International journal of breast cancer 2011: 595092. 22295229 10.4061/2011/595092 PMC3262577

[17] Fujishima Y, Nishiumi S, Masuda A, Inoue J, Nguyen NM, et al (2011) Autophagy in the intestinal epithelium reduces endotoxin-induced inflammatory responses by inhibiting NF-kappaB activation. Archives of biochemistry and biophysics 506: 223–235. 21156154 10.1016/j.abb.2010.12.009

[18] Lee KM, Hwang SK, Lee JA (2013) Neuronal Autophagy and Neurodevelopmental Disorders. Experimental neurobiology 22: 133–142. 24167408 10.5607/en.2013.22.3.133 PMC3807000

[19] Fimia GM, Kroemer G, Piacentini M (2013) Molecular mechanisms of selective autophagy. Cell death and differentiation 20: 1–2. 23222486 10.1038/cdd.2012.97 PMC3524629

[20] Klionsky DJ (2007) Autophagy: from phenomenology to molecular understanding in less than a decade. Nature reviews Molecular cell biology 8: 931–937. 17712358 10.1038/nrm2245

[21] Mehrpour M, Esclatine A, Beau I, Codogno P (2010) Overview of macroautophagy regulation in mammalian cells. Cell research 20: 748–762. 20548331 10.1038/cr.2010.82

[22] Choi AM, Ryter SW, Levine B (2013) Autophagy in human health and disease. The New England journal of medicine 368: 651–662. 23406030 10.1056/NEJMra1205406

[23] Johansen T, Lamark T (2011) Selective autophagy mediated by autophagic adapter proteins. Autophagy 7: 279–296. 21189453 10.4161/auto.7.3.14487 PMC3060413

[24] Artal-Martinez de Narvajas A, Gomez TS, Zhang JS, Mann AO, Taoda Y, et al (2013) Epigenetic regulation of autophagy by the methyltransferase G9a. Molecular and cellular biology 33: 3983–3993. 23918802 10.1128/MCB.00813-13 PMC3811684

[25] Li T, Cui ZB, Ke XX, Tan J, Li FF, et al (2011) Essential role for p53 and caspase-9 in DNA damaging drug-induced apoptosis in neuroblastoma IMR32 cells. DNA and cell biology 30: 1045–1050. 21612408 10.1089/dna.2011.1255

[26] Li T, Wang L, Ke XX, Gong XY, Wan JH, et al (2012) DNA-damaging drug-induced apoptosis sensitized by N-myc in neuroblastoma cells. Cell biology international 36: 331–337. 21929510 10.1042/CBI20110231

[27] Askin FB, Perlman EJ (1998) Neuroblastoma and peripheral neuroectodermal tumors. Am J Clin Pathol 109: S23–30. 9533746

[28] Shah S, Ravindranath Y (1998) Neuroblastoma. Indian journal of pediatrics 65: 691–705. 10773924 10.1007/BF02731044

[29] Bessho F (1999) Incidence of neuroblastoma. Lancet 353: 70. 10.1016/S0140-6736(05)74838-8 10023976

[30] Sridhar S, Al-Moallem B, Kamal H, Terrile M, Stallings RL (2013) New insights into the genetics of neuroblastoma. Molecular diagnosis & therapy 17: 63–69. 23329364 10.1007/s40291-013-0019-6

[31] Zhu S, Yan X, Xiang Z, Ding HF, Cui H (2013) Leflunomide reduces proliferation and induces apoptosis in neuroblastoma cells in vitro and in vivo. PloS one 8: e71555. 23977077 10.1371/journal.pone.0071555 PMC3743402

[32] Mezentseva NV, Yang J, Kaur K, Iaffaldano G, Remond MC, et al (2013) The histone methyltransferase inhibitor BIX01294 enhances the cardiac potential of bone marrow cells. Stem cells and development 22: 654–667. 22994322 10.1089/scd.2012.0181 PMC3564468

[33] Liu F, Chen X, Allali-Hassani A, Quinn AM, Wigle TJ, et al (2010) Protein lysine methyltransferase G9a inhibitors: design, synthesis, and structure activity relationships of 2,4-diamino-7-aminoalkoxy-quinazolines. Journal of medicinal chemistry 53: 5844–5857. 20614940 10.1021/jm100478y PMC2920043

[34] Liu F, Barsyte-Lovejoy D, Allali-Hassani A, He Y, Herold JM, et al (2011) Optimization of cellular activity of G9a inhibitors 7-aminoalkoxy-quinazolines. Journal of medicinal chemistry 54: 6139–6150. 21780790 10.1021/jm200903z PMC3171737

[35] Kubicek S, O'Sullivan RJ, August EM, Hickey ER, Zhang Q, et al (2007) Reversal of H3K9me2 by a small-molecule inhibitor for the G9a histone methyltransferase. Molecular cell 25: 473–481. 17289593 10.1016/j.molcel.2007.01.017

[36] Molenaar JJ, Koster J, Zwijnenburg DA, van Sluis P, Valentijn LJ, et al (2012) Sequencing of neuroblastoma identifies chromothripsis and defects in neuritogenesis genes. Nature 483: 589–593. 22367537 10.1038/nature10910

[37] Chen QR, Song YK, Wei JS, Bilke S, Asgharzadeh S, et al (2008) An integrated cross-platform prognosis study on neuroblastoma patients. Genomics 92: 195–203. 18598751 10.1016/j.ygeno.2008.05.014 PMC2562635

[38] Simon T, Haberle B, Hero B, von Schweinitz D, Berthold F (2013) Role of surgery in the treatment of patients with stage 4 neuroblastoma age 18 months or older at diagnosis. Journal of clinical oncology: official journal of the American Society of Clinical Oncology 31: 752–758. 23284039 10.1200/JCO.2012.45.9339

[39] Sharp SE, Gelfand MJ, Shulkin BL (2011) Pediatrics: diagnosis of neuroblastoma. Seminars in nuclear medicine 41: 345–353. 21803184 10.1053/j.semnuclmed.2011.05.001

[40] Lim S, Kaldis P (2013) Cdks, cyclins and CKIs: roles beyond cell cycle regulation. Development 140: 3079–3093. 23861057 10.1242/dev.091744

[41] Lee IH, Finkel T (2013) Metabolic regulation of the cell cycle. Current opinion in cell biology. 10.1016/j.ceb.2013.07.002 PMC3836844 23890700

[42] Wu J, Dang Y, Su W, Liu C, Ma H, et al (2006) Molecular cloning and characterization of rat LC3A and LC3B–two novel markers of autophagosome. Biochemical and biophysical research communications 339: 437–442. 16300744 10.1016/j.bbrc.2005.10.211

[43] Huang X, Bai HM, Chen L, Li B, Lu YC (2010) Reduced expression of LC3B-II and Beclin 1 in glioblastoma multiforme indicates a down-regulated autophagic capacity that relates to the progression of astrocytic tumors. Journal of clinical neuroscience: official journal of the Neurosurgical Society of Australasia 17: 1515–1519. 20863706 10.1016/j.jocn.2010.03.051

[44] Meyer G, Czompa A, Reboul C, Csepanyi E, Czegledi A, et al. (2013) The Cellular Autophagy Markers Beclin-1 and LC3B-II are Increased during Reperfusion in Fibrillated Mouse Hearts. Current pharmaceutical design. 10.2174/138161281939131127122510 23590156

[45] Tait SW, Green DR (2013) Mitochondrial regulation of cell death. Cold Spring Harbor perspectives in biology 5. 10.1101/cshperspect.a008706 PMC3753705 24003207

[46] Barta T, Dolezalova D, Holubcova Z, Hampl A (2013) Cell cycle regulation in human embryonic stem cells: links to adaptation to cell culture. Experimental biology and medicine 238: 271–275. 23598972 10.1177/1535370213480711

[47] Mai S, Muster B, Bereiter-Hahn J, Jendrach M (2012) Autophagy proteins LC3B, ATG5 and ATG12 participate in quality control after mitochondrial damage and influence lifespan. Autophagy 8: 47–62. 22170153 10.4161/auto.8.1.18174 PMC3335991

[48] Hale AN, Ledbetter DJ, Gawriluk TR, Rucker EB 3rd (2013) Autophagy: regulation and role in development. Autophagy 9: 951–972. 24121596 10.4161/auto.24273 PMC3722331

[49] Zhao H, Yang M, Zhao J, Wang J, Zhang Y, et al (2013) High expression of LC3B is associated with progression and poor outcome in triple-negative breast cancer. Medical oncology 30: 475. 23371253 10.1007/s12032-013-0475-1

[50] Lu Z, Tian Y, Salwen HR, Chlenski A, Godley LA, et al (2013) Histone-lysine methyltransferase EHMT2 is involved in proliferation, apoptosis, cell invasion, and DNA methylation of human neuroblastoma cells. Anti-cancer drugs 24: 484–493. 23466651 10.1097/CAD.0b013e32835ffdbb PMC3649845

[51] Kim Y, Kim YS, Kim DE, Lee JS, Song JH, et al (2013) BIX-01294 induces autophagy-associated cell death via EHMT2/G9a dysfunction and intracellular reactive oxygen species production. Autophagy 9: 2126–2139. 24322755 10.4161/auto.26308

