# Correction: Inhibition of H3K9 Methyltransferase G9a Repressed Cell Proliferation and Induced Autophagy in Neuroblastoma Cells

**DOI:** 10.1371/journal.pone.0213135

**Published:** 2019-02-26

**Authors:** Xiao-Xue Ke, Dunke Zhang, Shunqin Zhu, Qingyou Xia, Zhonghuai Xiang, Hongjuan Cui

The Data Availability Statement for this article [1] is incorrect. All relevant data are not provided in the paper and its Supporting Information files. The authors have provided the primary data underlying the charts, the available original microscopy image files, and the available processed flow cytometry data in Supporting Information files, S1–S3 Files. Additionally, images of mice and excised tumors from the tumorigenicity experiments in Figure 5C and D are provided. Complete individual-level tumor volume and weight data are provided. The authors acknowledge that there are some potential inconsistencies between the tumor volume and tumor weight data, which may have been caused by random error in taking caliper measurements in live mice. Additional methodological information regarding the experimental design for the *in vivo* tumor studies is provided by the authors as follows:

The study design incorporated humane endpoint criteria, and steps were taken to minimize the suffering of the animals. The physical condition of the animals was monitored throughout the experiment period, including the body weight, behaviors, etc. To determine the endpoint, four criteria were applied: 1, the tumor diameter over 30 mm, or tumor weight exceeds 4g; 2, the mice are sick, bad spirit, extremely thin; 3, the tumor-bearing mice have difficulty in moving; 4, the experiment period exceeds 45 days. According to these criteria, BE(2)-C groups were euthanized due to the tumor diameter over 30 mm, while AS groups were euthanized due to reduced activity and movement of the mice.

Note that the original microscopy image files are available for only one of the three independent experiments described in Figures 4A and 7A; the processed flow cytometry data are available for only one of three independent experiments described in Figure 3A. The raw flow cytometry data are not available. The original uncropped Western blot images underlying Figures 1G, 3C, 4C-D, 6A, 6E, and 7C-D are not available.

## Supporting information

S1 File Dataset. (ZIP)

S2 File Microscopy image data (Part 1). (ZIP)

S3 File Microscopy image data (Part 2). (ZIP)
