# Supplementary figures and images for: Inhibition of H3K9 Methyltransferase G9a Repressed Cell Proliferation and Induced Autophagy in Neuroblastoma Cells
Source: PLoS One. 2014 Sep 8;9(9):e106962. doi: 10.1371/journal.pone.0106962 (PMC4157855; doi:10.1371/journal.pone.0106962)

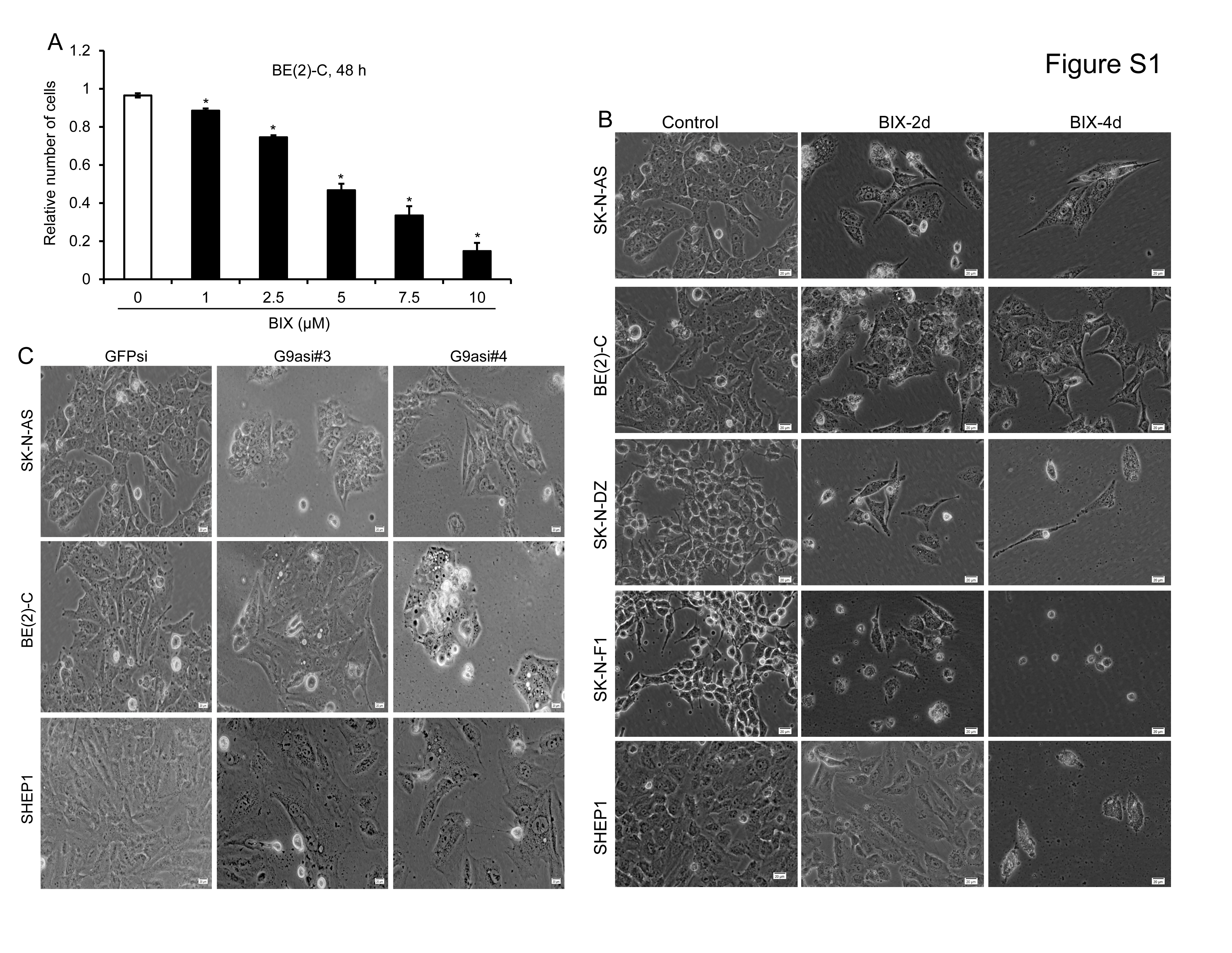

Supplement: Figure S1 — Inhibition of G9a suppresses neuroblastoma cell proliferation. A, neuroblastoma BE(2)-C cells were treated with BIX01294 for 48 h at concentrations of 1, 2.5, 5, 7.5, and 10 µM. Proliferation was determined by trypan blue exclusion to differentiate between dead and live cells. Error bars, SD, n = 5. Statistical analysis was performed using two-tailed student's t-test, *p≤0.01. B, morphologic examination of five neuroblastoma cells treated with 5 µM BIX01294 or water for 2 and 4 days. Scale bars, 20 µm. C, morphological examination of three neuroblastoma cell lines express GFPsi, G9asi#3 or G9asi#4. Cells expressing GFPsi are shown as the biological control. Scale bar, 20 µm. (TIF) [file pone.0106962.s001.tif]

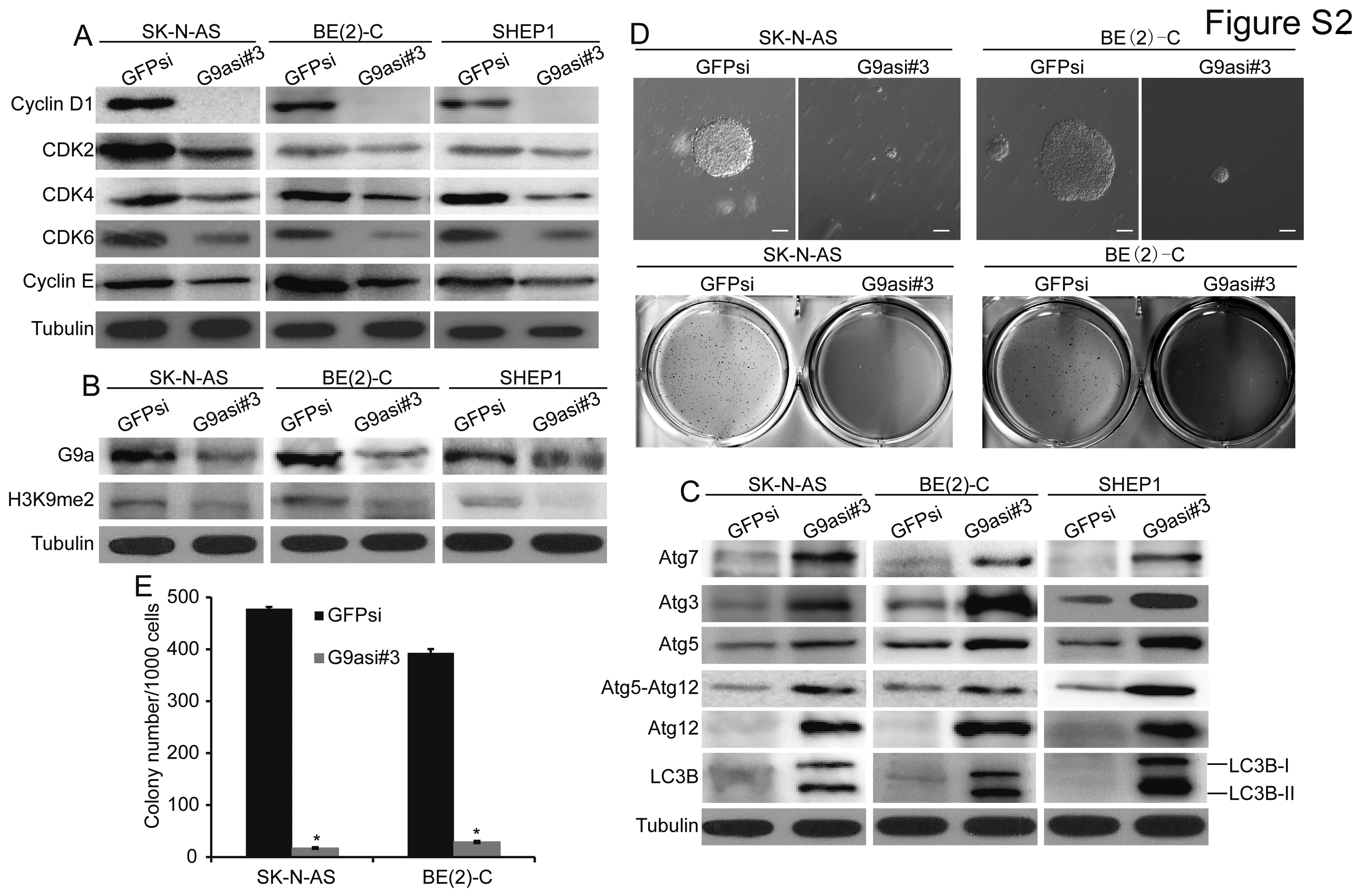

Supplement: Figure S2 — Downregulation of G9a represses neuroblastoma cell proliferation and tumorigenicity and induces autophagy. A, B and C, western blot analysis of cyclins and CDKs associated with the G1 phase (A), G9a function (B) and autophagy-related genes (C) in neuroblastoma cells expressing GFPsi or G9asi#3. α-Tubulin levels are shown as the loading control. Cells expressing GFPsi are shown as the biological control. D, neuroblastoma cells were plated at 1×10^3^ cells per well in six-well culture plates. After 14 to 21 days of culture, soft agar colonies grown with cells expressing GFPsi. As shown, the cells with G9a knockdown were observed to give rise to small and scanty colonies in soft agar, Scale bars, 50 µm. E, colonies that were larger than 0.5 mm or that contained more than 50 cells were recorded. Each column represents the average obtained from three independent experiments; error bars, SD. Statistical analysis was performed using two-tailed student's t-test, *p≤0.01. (TIF) [file pone.0106962.s002.tif]
