# Supplementary figures and images for: Correction: Inhibition of H3K9 Methyltransferase G9a Repressed Cell Proliferation and Induced Autophagy in Neuroblastoma Cells
Source: PLoS One. 2019 Feb 26;14(2):e0213135. doi: 10.1371/journal.pone.0213135 (PMC6391043; doi:10.1371/journal.pone.0213135)

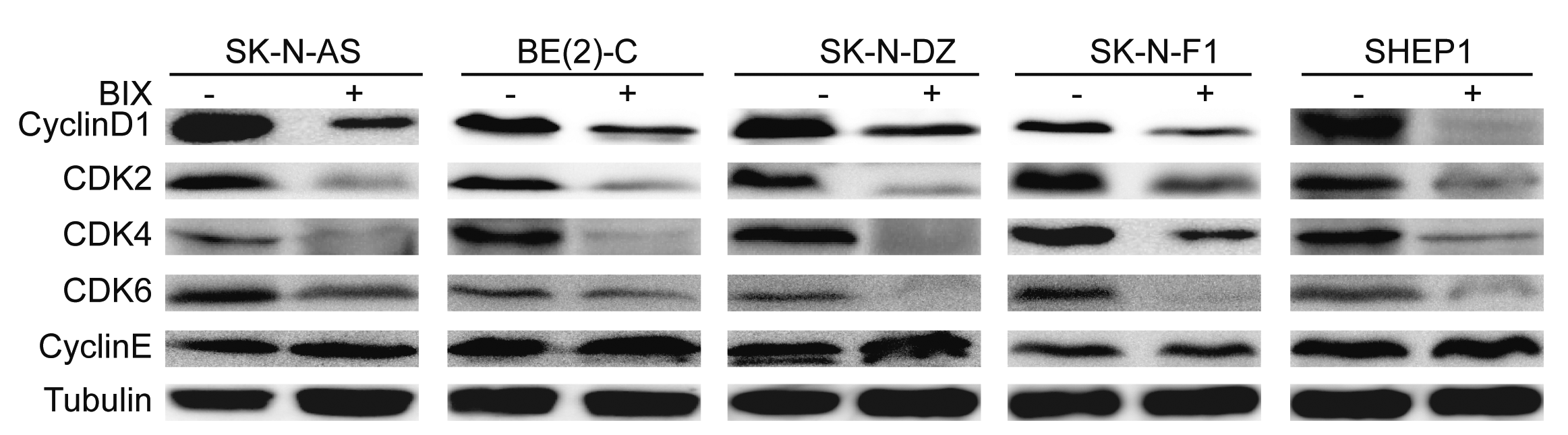

Supplement: S1 File — (ZIP) [file pone.0213135.s001.zip › S1_File/fig3C-cell cycle-blot.tif]

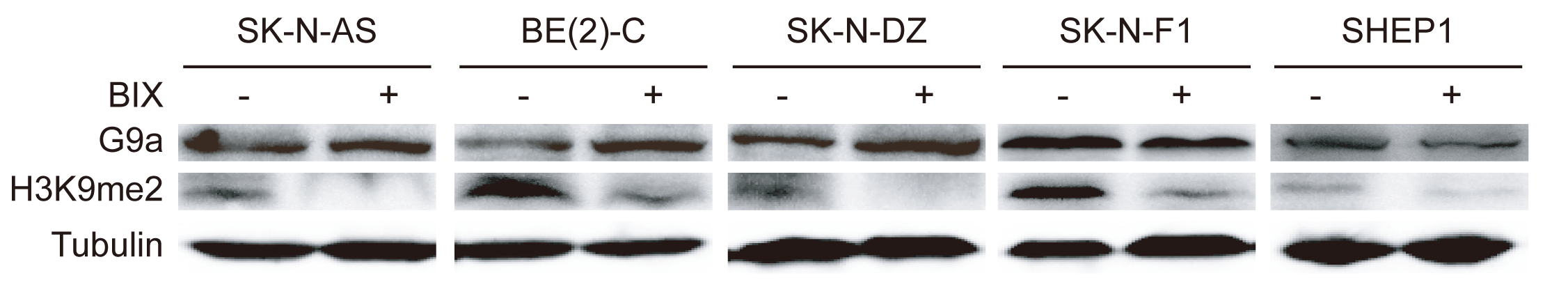

Supplement: S1 File — (ZIP) [file pone.0213135.s001.zip › S1_File/fig4C-H3K9-blot.tif]

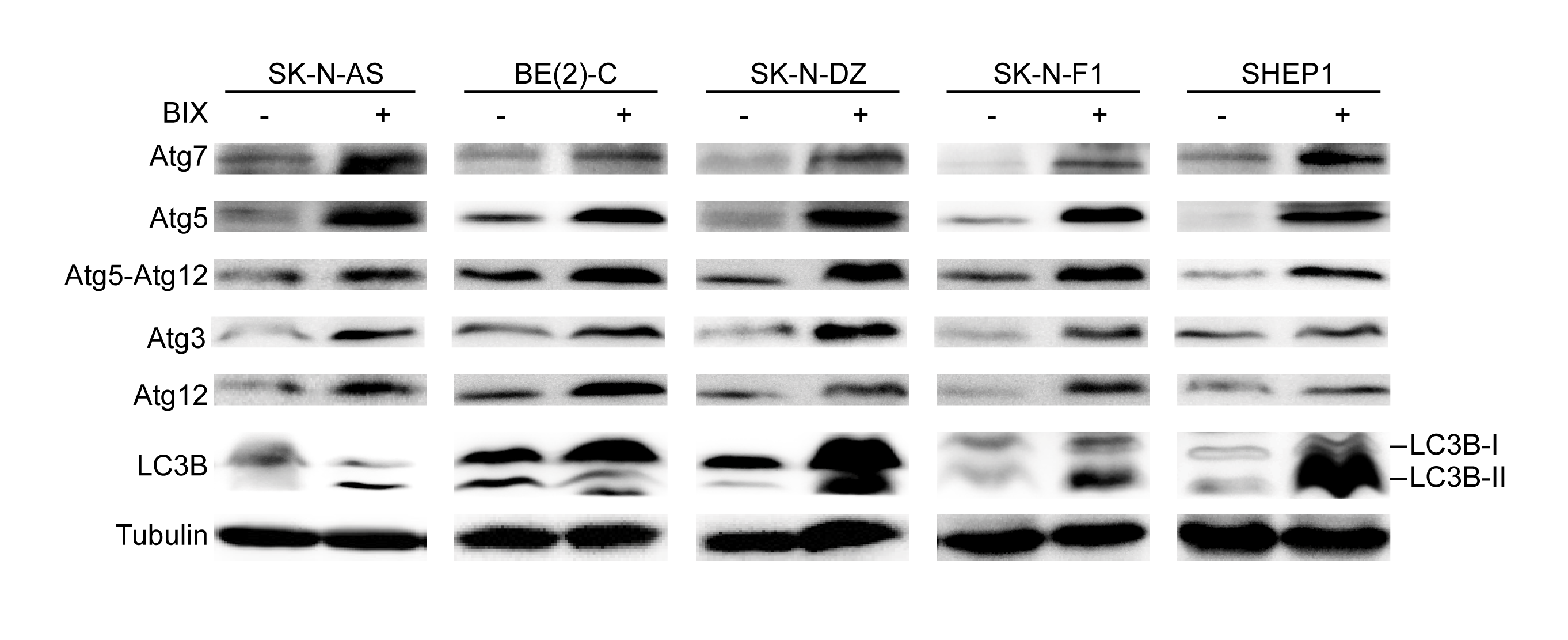

Supplement: S1 File — (ZIP) [file pone.0213135.s001.zip › S1_File/fig4D-ATG-blot.tif]

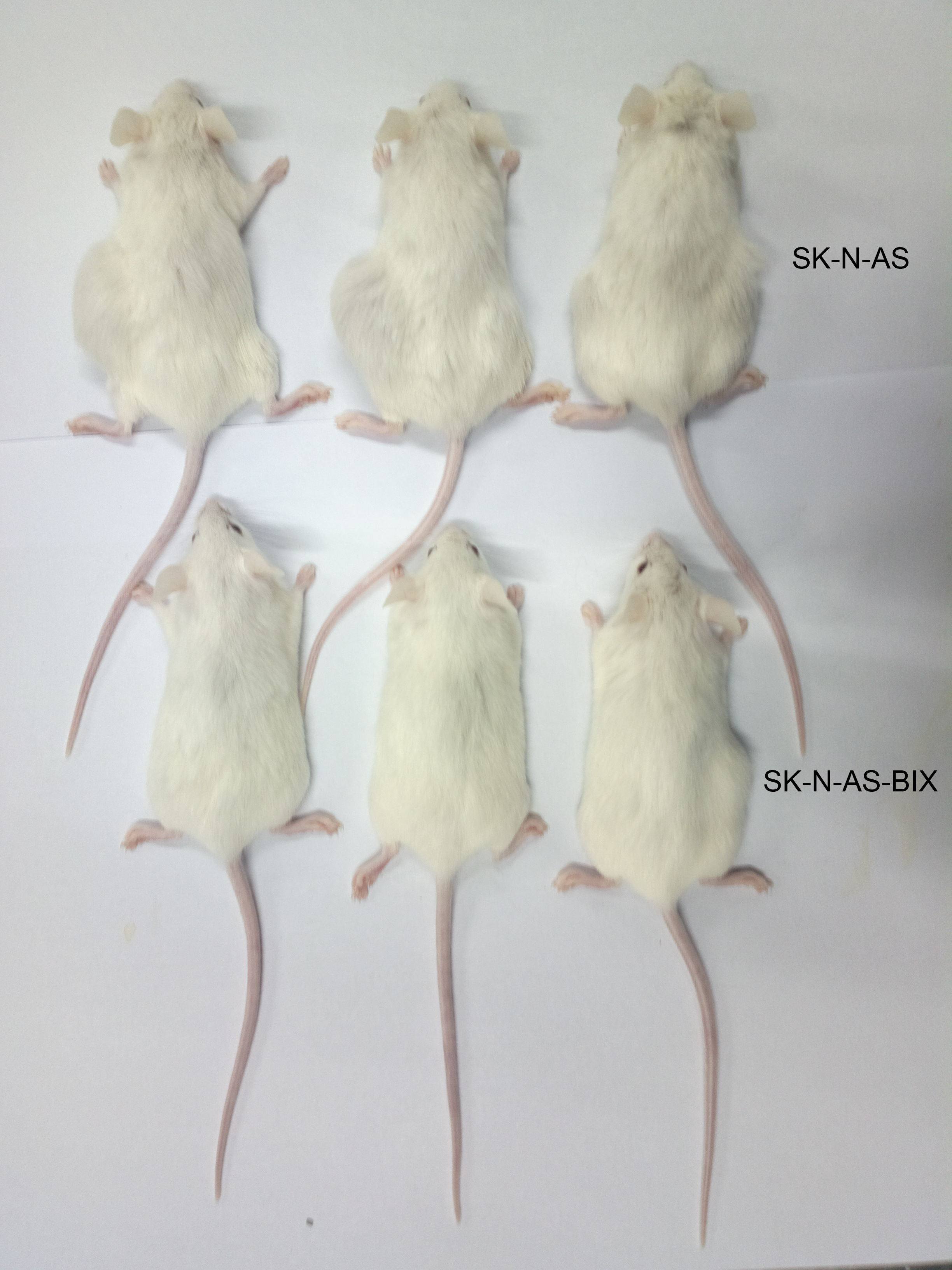

Supplement: S1 File — (ZIP) [file pone.0213135.s001.zip › S1_File/fig5C、D-tumor-AS-mice photo.tif]

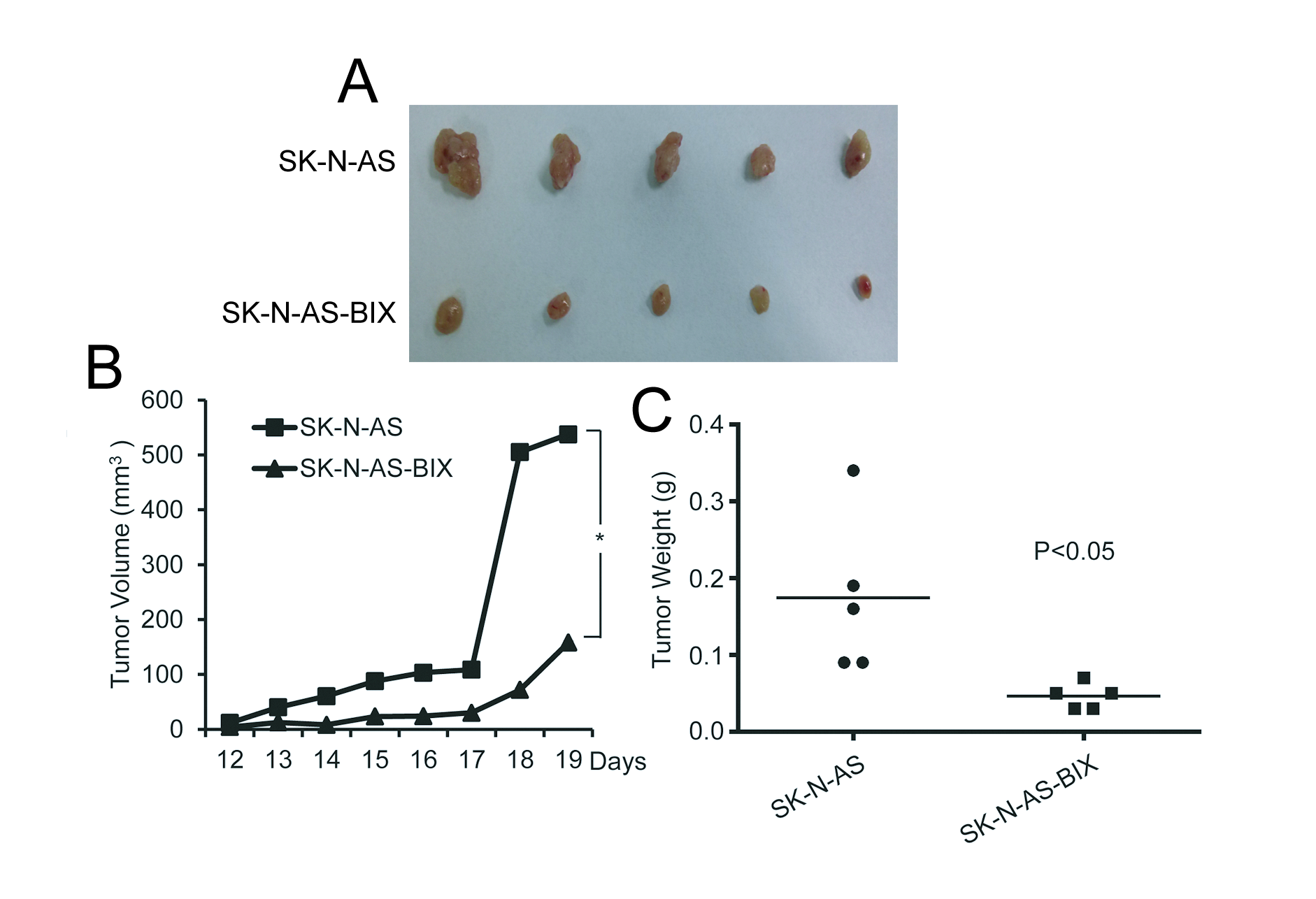

Supplement: S1 File — (ZIP) [file pone.0213135.s001.zip › S1_File/fig5C、D-tumor-AS.tif]

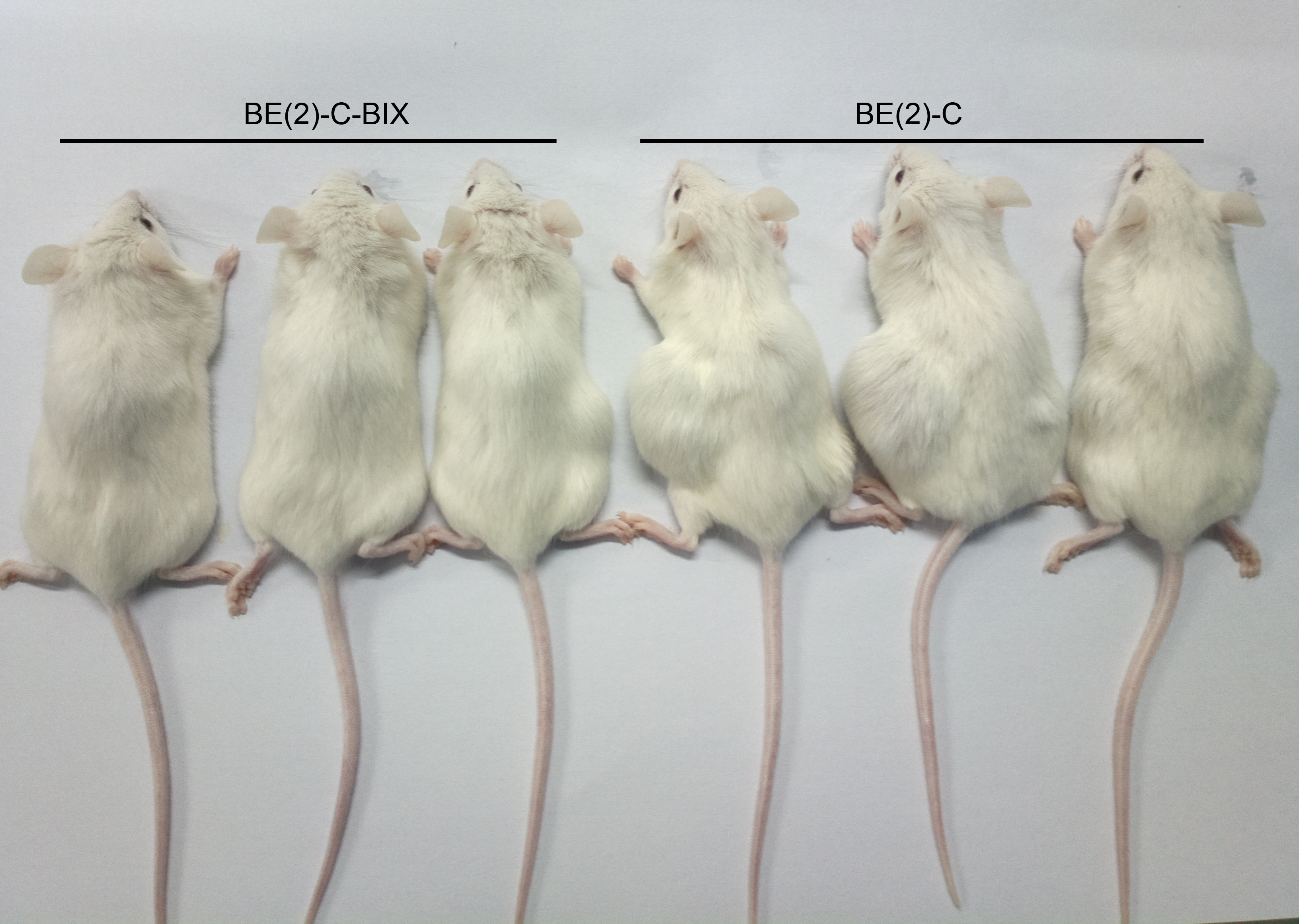

Supplement: S1 File — (ZIP) [file pone.0213135.s001.zip › S1_File/fig5C、D-tumor-BE2C-mice photo.tif]

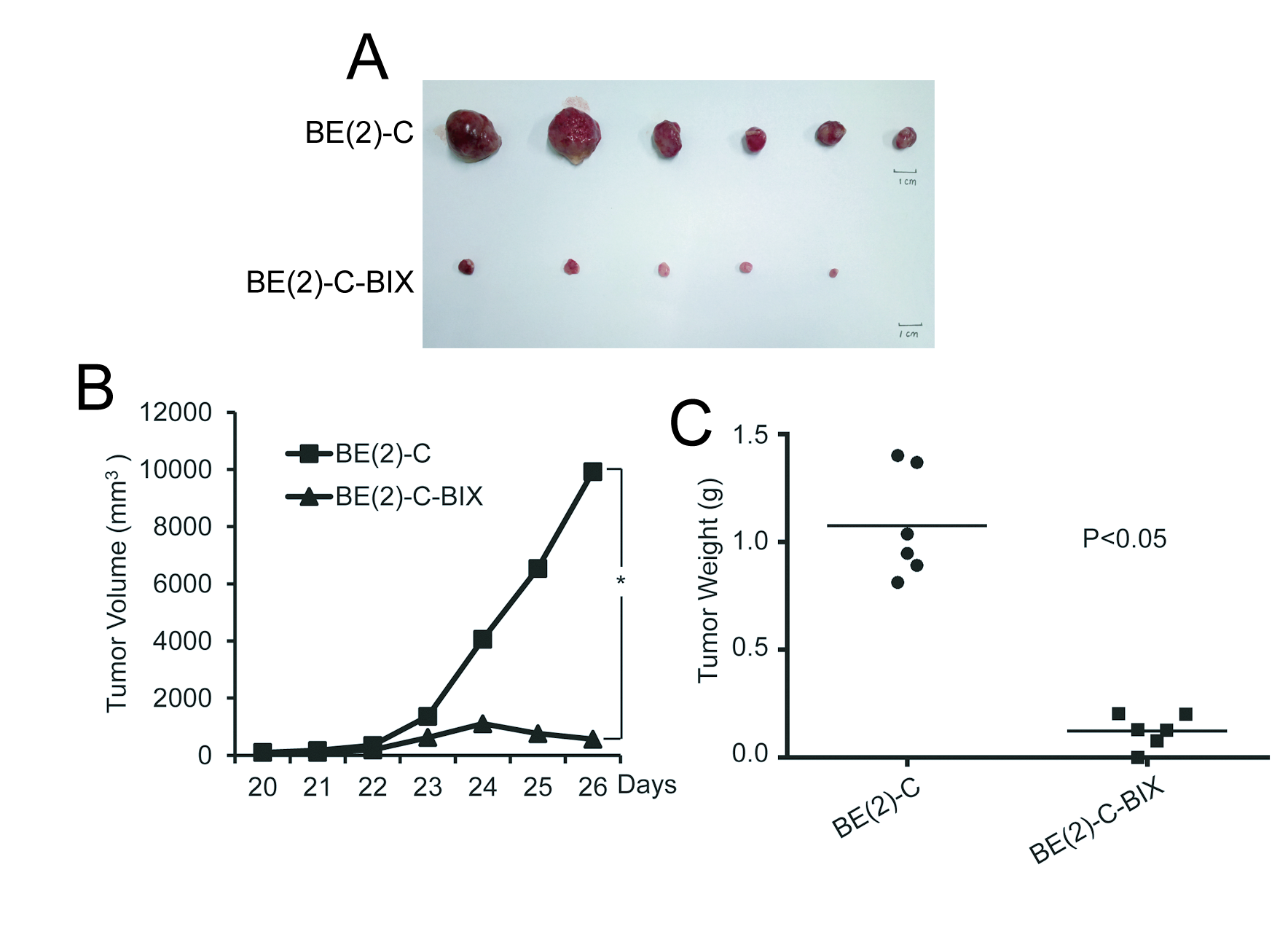

Supplement: S1 File — (ZIP) [file pone.0213135.s001.zip › S1_File/fig5C、D-tumor-BE2C.tif]

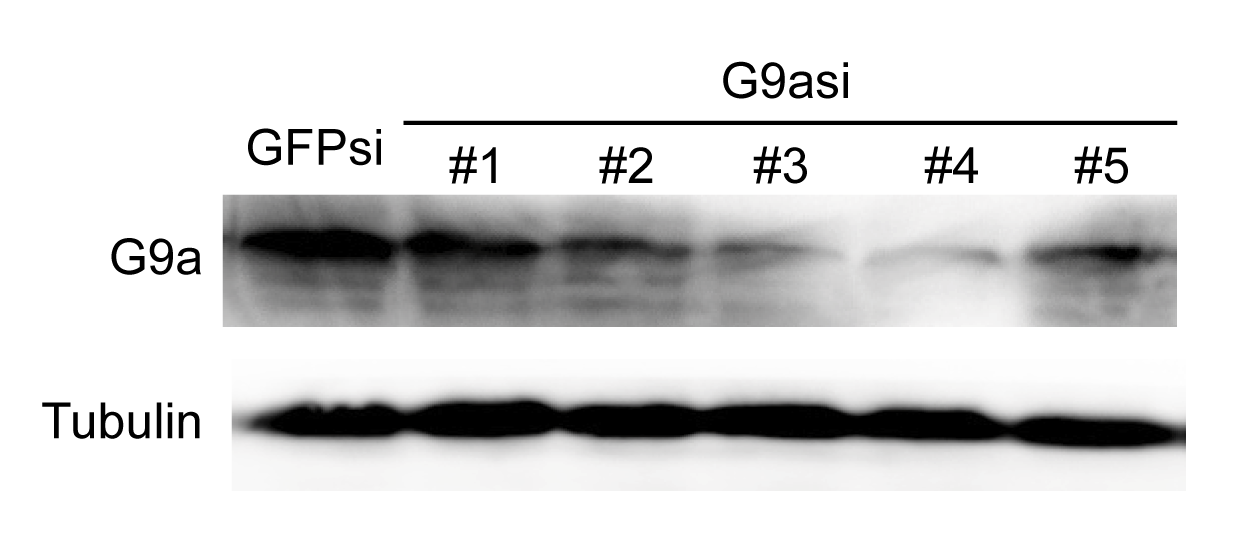

Supplement: S1 File — (ZIP) [file pone.0213135.s001.zip › S1_File/fig6A-G9asi-blot.tif]

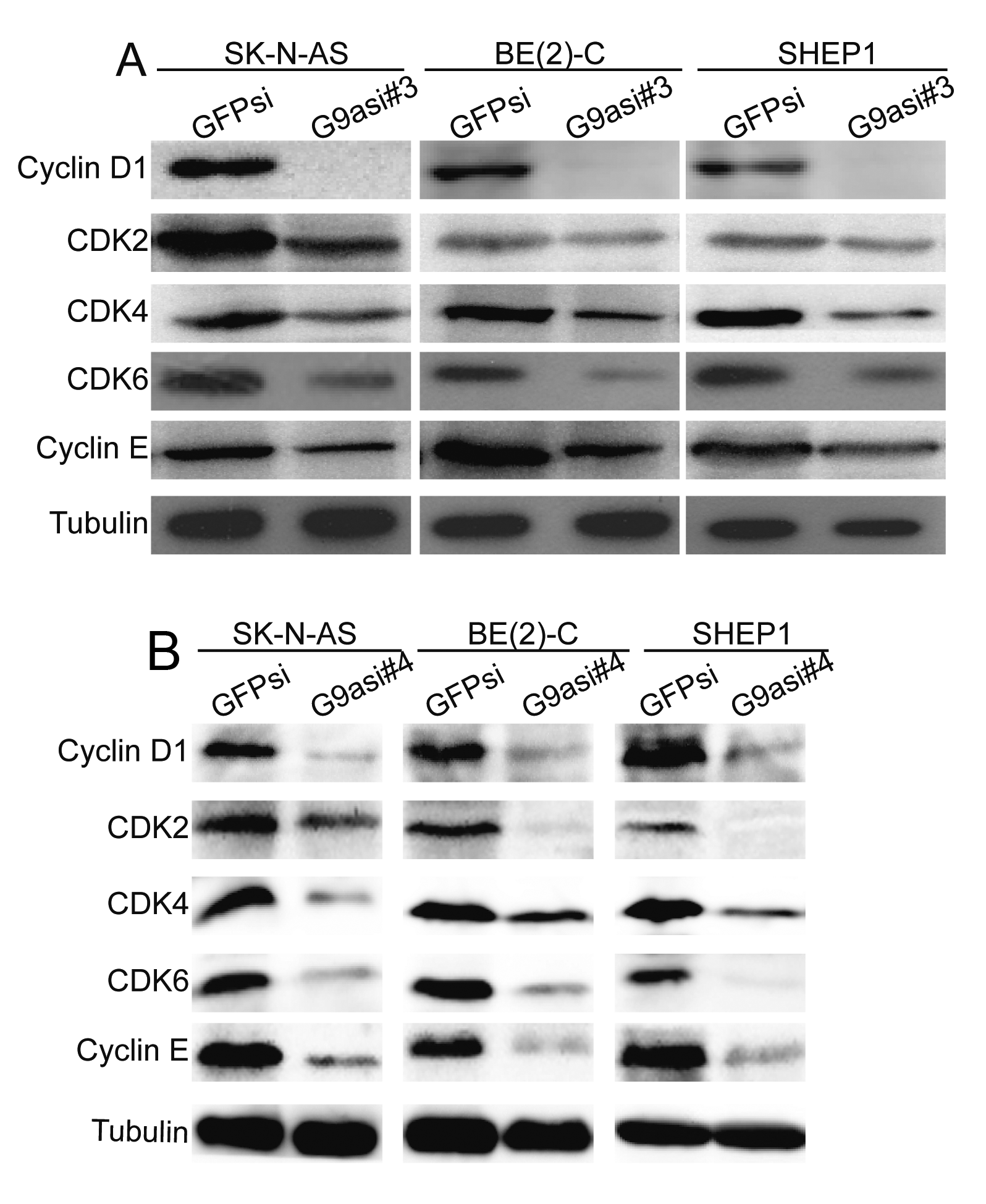

Supplement: S1 File — (ZIP) [file pone.0213135.s001.zip › S1_File/fig6E-figS2A-cell cycle-G9asi-blot.tif]

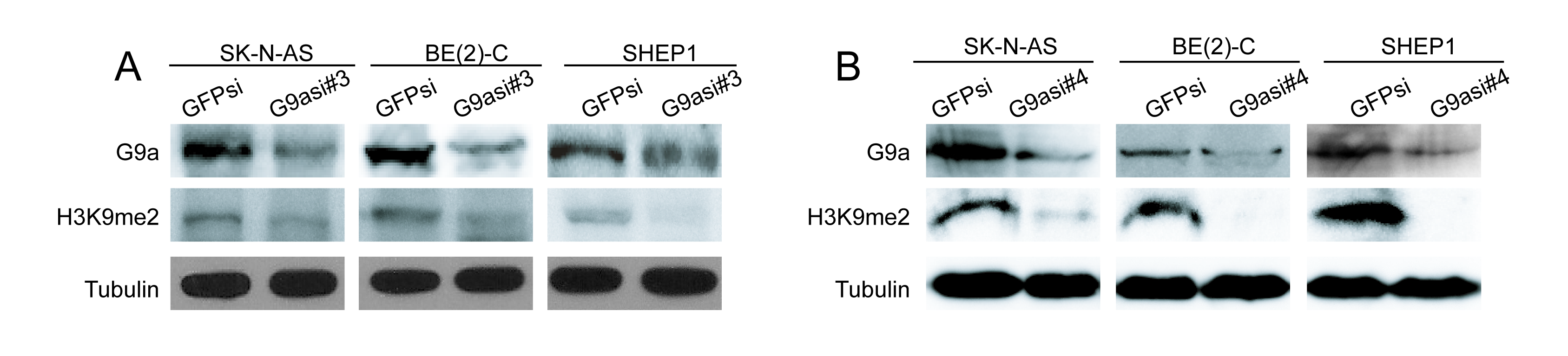

Supplement: S1 File — (ZIP) [file pone.0213135.s001.zip › S1_File/fig7C-figS2B-H3K9-G9asi-blot.tif]

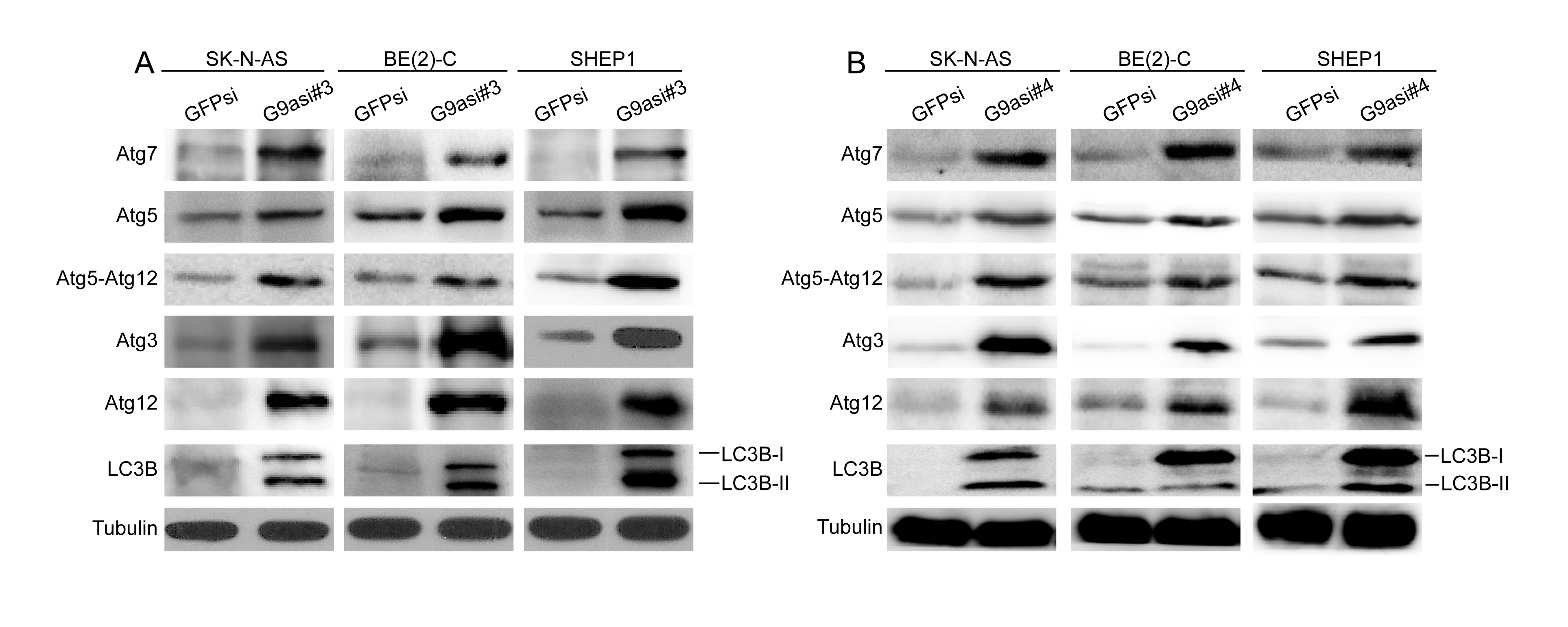

Supplement: S1 File — (ZIP) [file pone.0213135.s001.zip › S1_File/fig7D-figS2C-ATG-G9asi-blot.tif]
