# Supplementary material for: Correction: Inhibition of H3K9 Methyltransferase G9a Repressed Cell Proliferation and Induced Autophagy in Neuroblastoma Cells
Source: PLoS One. 2019 Feb 26;14(2):e0213135. doi: 10.1371/journal.pone.0213135 (PMC6391043; doi:10.1371/journal.pone.0213135)
Supplement: S1 File — (ZIP) [file pone.0213135.s001.zip › S1_File/Flow Cytometry Data.doc]

AS-BIX

File analyzed: cycl-120605.019

Date analyzed: 12433856--12435756

Model: 1nn0n_DSF

Analysis type: Manual analysis

Diploid: 100.00 %

Dip G1: 70.76 % at 45.69

Dip G2: 7.94 % at 93.63

Dip S: 21.30 %	G2/G1: 2.05

%CV: 6.16

Total S-Phase: 21.30 %

Total B.A.D.: 0.00 %	no debris no aggs

Debris: %

Aggregates: 0.00 %

Modeled events: 24478

All cycle events: 24478

Cycle events per channel: 500

RCS: 4.254

AS-control

File analyzed: cycl-120605.022

Date analyzed: 12433856--12435756

Model: 1nn0n_DSF

Analysis type: Manual analysis

Diploid: 100.00 %

Dip G1: 61.86 % at 46.76

Dip G2: 5.33 % at 98.00

Dip S: 32.81 %	G2/G1: 2.10

%CV: 6.72

Total S-Phase: 32.81 %

Total B.A.D.: 0.00 %	no debris no aggs

Debris: %

Aggregates: 0.00 %

Modeled events: 24945

All cycle events: 24945

Cycle events per channel: 477

RCS: 8.766

BE2C-BIX

File analyzed: cycl-120605.001

Date analyzed: 12433856--12435756

Model: 1nn0n_DSF

Analysis type: Manual analysis

Diploid: 100.00 %

Dip G1: 53.69 % at 38.75

Dip G2: 5.27 % at 88.35

Dip S: 41.04 %	G2/G1: 2.28

%CV: 18.78

Total S-Phase: 41.04 %

Total B.A.D.: 0.00 %	no debris no aggs

Debris: %

Aggregates: 0.00 %

Modeled events: 15552

All cycle events: 15552

Cycle events per channel: 307

RCS: 13.240

BE2C-control

File analyzed: cycl-120605.005

Date analyzed: 12433856--12435756

Model: 1nn0n_DSF

Analysis type: Manual analysis

Diploid: 100.00 %

Dip G1: 41.69 % at 45.98

Dip G2: 0.06 % at 102.44

Dip S: 58.24 %	G2/G1: 2.23

%CV: 9.54

Total S-Phase: 58.24 %

Total B.A.D.: 0.00 %	no debris no aggs

Debris: %

Aggregates: 0.00 %

Modeled events: 19755

All cycle events: 19755

Cycle events per channel: 344

RCS: 11.863

DZ-BIX

File analyzed: cycl-120605.017

Date analyzed: 12433856--12435756

Model: 1nn0n_DSF

Analysis type: Manual analysis

Diploid: 100.00 %

Dip G1: 67.94 % at 46.13

Dip G2: 5.87 % at 91.41

Dip S: 26.19 %	G2/G1: 1.98

%CV: 7.38

Total S-Phase: 26.19 %

Total B.A.D.: 0.00 %	no debris no aggs

Debris: %

Aggregates: 0.00 %

Modeled events: 24003

All cycle events: 24003

Cycle events per channel: 519

RCS: 4.783

DZ-control

File analyzed: cycl-120605.013

Date analyzed: 12433856--12435756

Model: 1nn0n_DSF

Analysis type: Manual analysis

Diploid: 100.00 %

Dip G1: 51.83 % at 40.44

Dip G2: 1.23 % at 83.19

Dip S: 46.95 %	G2/G1: 2.06

%CV: 7.55

Total S-Phase: 46.95 %

Total B.A.D.: 0.00 %	no debris no aggs

Debris: %

Aggregates: 0.00 %

Modeled events: 24155

All cycle events: 24155

Cycle events per channel: 552

RCS: 10.524

F1-BIX

File analyzed: cycl-120620.005

Date analyzed: 12313552--12315452

Model: 1nn0n_DSF

Analysis type: Manual analysis

Diploid: 100.00 %

Dip G1: 64.47 % at 44.44

Dip G2: 25.76 % at 72.56

Dip S: 9.77 %	G2/G1: 1.63

%CV: 15.96

Total S-Phase: 9.77 %

Total B.A.D.: 0.00 %	no debris no aggs

Debris: %

Aggregates: 0.00 %

Modeled events: 22488

All cycle events: 22488

Cycle events per channel: 772

RCS: 4.724

F1-control

File analyzed: cycl-120620.001

Date analyzed: 12313552--12315452

Model: 1nn0n_DSF

Analysis type: Manual analysis

Diploid: 100.00 %

Dip G1: 48.03 % at 42.38

Dip G2: 15.16 % at 82.42

Dip S: 36.81 %	G2/G1: 1.94

%CV: 10.98

Total S-Phase: 36.81 %

Total B.A.D.: 0.00 %	no debris no aggs

Debris: %

Aggregates: 0.00 %

Modeled events: 17677

All cycle events: 17677

Cycle events per channel: 431

RCS: 9.151

SHEP1-BIX

File analyzed: cycl-120605.012

Date analyzed: 12433856--12435756

Model: 1nn0n_DSF

Analysis type: Manual analysis

Diploid: 100.00 %

Dip G1: 81.90 % at 45.83

Dip G2: 4.54 % at 92.85

Dip S: 13.56 %	G2/G1: 2.03

%CV: 4.90

Total S-Phase: 13.56 %

Total B.A.D.: 0.00 %	no debris no aggs

Debris: %

Aggregates: 0.00 %

Modeled events: 24683

All cycle events: 24683

Cycle events per channel: 514

RCS: 4.924

SHEP1-control

File analyzed: cycl-120605.007

Date analyzed: 12433856--12435756

Model: 1nn0n_DSF

Analysis type: Manual analysis

Diploid: 100.00 %

Dip G1: 67.86 % at 47.10

Dip G2: 10.73 % at 94.93

Dip S: 21.41 %	G2/G1: 2.02

%CV: 5.19

Total S-Phase: 21.41 %

Total B.A.D.: 0.00 %	no debris no aggs

Debris: %

Aggregates: 0.00 %

Modeled events: 27624

All cycle events: 27624

Cycle events per channel: 566

RCS: 3.788
